# Unleashing novel horizons in advanced prostate cancer treatment: investigating the potential of prostate specific membrane antigen-targeted nanomedicine-based combination therapy

**DOI:** 10.3389/fimmu.2023.1265751

**Published:** 2023-09-19

**Authors:** Mingze He, Yu Cao, Changliang Chi, Jiang Zhao, Eunice Chong, Ke Xin Casey Chin, Nicole Zian Vi Tan, Korolev Dmitry, Guodong Yang, Xinyi Yang, Kebang Hu, Mikhail Enikeev

**Affiliations:** ^1^ Institute for Urology and Reproductive Health, I.M. Sechenov First Moscow State Medical University (Sechenov University), Moscow, Russia; ^2^ I.M. Sechenov First Moscow State Medical University (Sechenov University), Moscow, Russia; ^3^ Department of Urology, First Hospital of Jilin University, Changchun, China; ^4^ Department of Urology, Xi’an First Hospital, Xi’an, China

**Keywords:** prostate specific membrane antigen, nano delivery system, advanced prostate cancer, theragnostics, multimodal combination therapy

## Abstract

Prostate cancer (PCa) is a prevalent malignancy with increasing incidence in middle-aged and older men. Despite various treatment options, advanced metastatic PCa remains challenging with poor prognosis and limited effective therapies. Nanomedicine, with its targeted drug delivery capabilities, has emerged as a promising approach to enhance treatment efficacy and reduce adverse effects. Prostate-specific membrane antigen (PSMA) stands as one of the most distinctive and highly selective biomarkers for PCa, exhibiting robust expression in PCa cells. In this review, we explore the applications of PSMA-targeted nanomedicines in advanced PCa management. Our primary objective is to bridge the gap between cutting-edge nanomedicine research and clinical practice, making it accessible to the medical community. We discuss mainstream treatment strategies for advanced PCa, including chemotherapy, radiotherapy, and immunotherapy, in the context of PSMA-targeted nanomedicines. Additionally, we elucidate novel treatment concepts such as photodynamic and photothermal therapies, along with nano-theragnostics. We present the content in a clear and accessible manner, appealing to general physicians, including those with limited backgrounds in biochemistry and bioengineering. The review emphasizes the potential benefits of PSMA-targeted nanomedicines in enhancing treatment efficiency and improving patient outcomes. While the use of PSMA-targeted nano-drug delivery has demonstrated promising results, further investigation is required to comprehend the precise mechanisms of action, pharmacotoxicity, and long-term outcomes. By meticulous optimization of the combination of nanomedicines and PSMA ligands, a novel horizon of PSMA-targeted nanomedicine-based combination therapy could bring renewed hope for patients with advanced PCa.

## 1 Introduction

Prostate cancer (PCa) is a highly prevalent malignancy among middle-aged and older men, with the incidence increasing each year in recent years (1, 2). Based on the risk stratification provided by the European Urology Association (EUA), patients with low-risk PCa can undergo active surveillance without surgical intervention (3). For early-stage localized PCa, radical prostatectomy and postoperative radiotherapy are the treatment of choice (4). Androgen deprivation therapy (ADT) along or combined with chemotherapy or radiotherapy depends on the individual condition is considered as the first-line therapy for advanced PCa (5, 6). However, the effectiveness of ADT for advanced metastatic PCa is only temporary and is associated with numerous multisystemic side effects, while the five-year survival rate is only approximately 30% and the expected life expectancy is less than 20 months (7–11). Over time, the effects of ADT fade, and most patients progress to castration-resistant PCa (CRPC) (12). According to EUA guideline, CRPC is defined as a castrate serum testosterone < 50 ng/dL or 1.7 nmol/L plus either biochemical progression including repeated measurements of prostate specific antigen (PSA) or radiological progression according to RECIST (Response Evaluation Criteria in Solid Tumors) (13). Despite several clinical trials are currently extended in order to evaluate the efficacy of selected anti-androgen, chemotherapeutic and immunotherapeutic agents (14–17), the metastatic CRPC (mCRPC) currently has no cure with poor prognosis; hence, the patient’s quality of life is highly concerning (18, 19).

In recent times, nanomedicine has garnered significant attention for its potential in addressing solid tumors. It is heralded for its distinctive benefits, including targeted drug delivery and the capacity for on-demand adjustments in physicochemical, pharmacokinetic, and pharmacodynamic attributes. This encompasses aspects such as solubility, stability, and circulation time within the bloodstream. These capabilities enable precise control over drug release and transportation, thereby enhancing effectiveness, diminishing drug-related adverse effects, and mitigating multidrug resistance (MDR) during chemotherapy (20–22). Therefore, it is believed that implantation of the drug-nanoparticles (NP) delivery system containing the engaged therapeutic agents with a variety of pH-responsive, enzyme-responsive and redox-responsive nanomedical-materials will drastically enhance the effectiveness of multimodal therapies.

In the current stage of practice, active and passive targeting are being utilized to achieve selectivity and specificity in tumor cells. Passive delivery aims to enhance the permeability and retention effect (EPR-effect) of the NP in the tumor microenvironment (TME) (23). However, Yuko et al. (24) indicated that relying solely on EPR for delivering nanodrug results in a less than 2-fold increase in drug delivery and concentration, which is insufficient in treating most cancers effectively. In such cases, the use of active targeting of cell surface receptors such as prostate specific membrane antigen (PSMA) appears to be more practical and effective.

PSMA is a type II transmembrane protein containing glutamate carboxypeptidase (25). It is mainly expressed in prostate, kidney, salivary gland, nervous system glial, and small intestine jejunal brush border (26). However, it is overexpressed in PCa cells, and its expression increases along with the Gleason score (GS) and tumor malignancy, especially in CRPC (27–29). Meanwhile, PSMA is also expressed in the neovascular system during malignant angiogenesis (30), therefore it is currently considered as the promising tumor associated antigen (TAA) and are engaged in multimodal combination therapy of PCa. In addition, PSMA-specific binding ligands can be internalized by a particular sequence motif within the PSMA structural domain through endocytosis to enhance the therapeutic dose uptake in the tumor cells (27, 28). Hence, conducting PSMA-targeted NPs is of high anticipation. In addition, a related study found that 88% of patients with recurrent PCa had PSMA-positive (PSMA^+^) phenotypes, suggesting that most patients with mCRPC may be relatively sensitive and responsive to PSMA-targeted therapy (31).

This review aimed to explore the diverse applications of PSMA-targeted NP in the advanced PCa management from a cross-disciplinary perspective. Our primary objective is to facilitate the integration of cutting-edge nanomedicine research into clinical practice, making it accessible to the broader medical community. Therefore, we have discussed the mainstream treatment strategies in advanced PCa, encompassing chemotherapy, radiotherapy, and immunotherapy, in the context of PSMA-targeted nanomedicines. Furthermore, we have briefly elucidated the state-of-the-art in novel treatment concepts, such as photodynamic and photothermal therapies, along with nano-theragnostics. This review is tailored to appeal to general physicians with a keen interest in this emerging field, even those with limited backgrounds in biochemistry and bioengineering, as we have presented the content in a straightforward manner to avoid unnecessary complexity.

## 2 Nano-materials and their basic working principles

**Figure 1:**
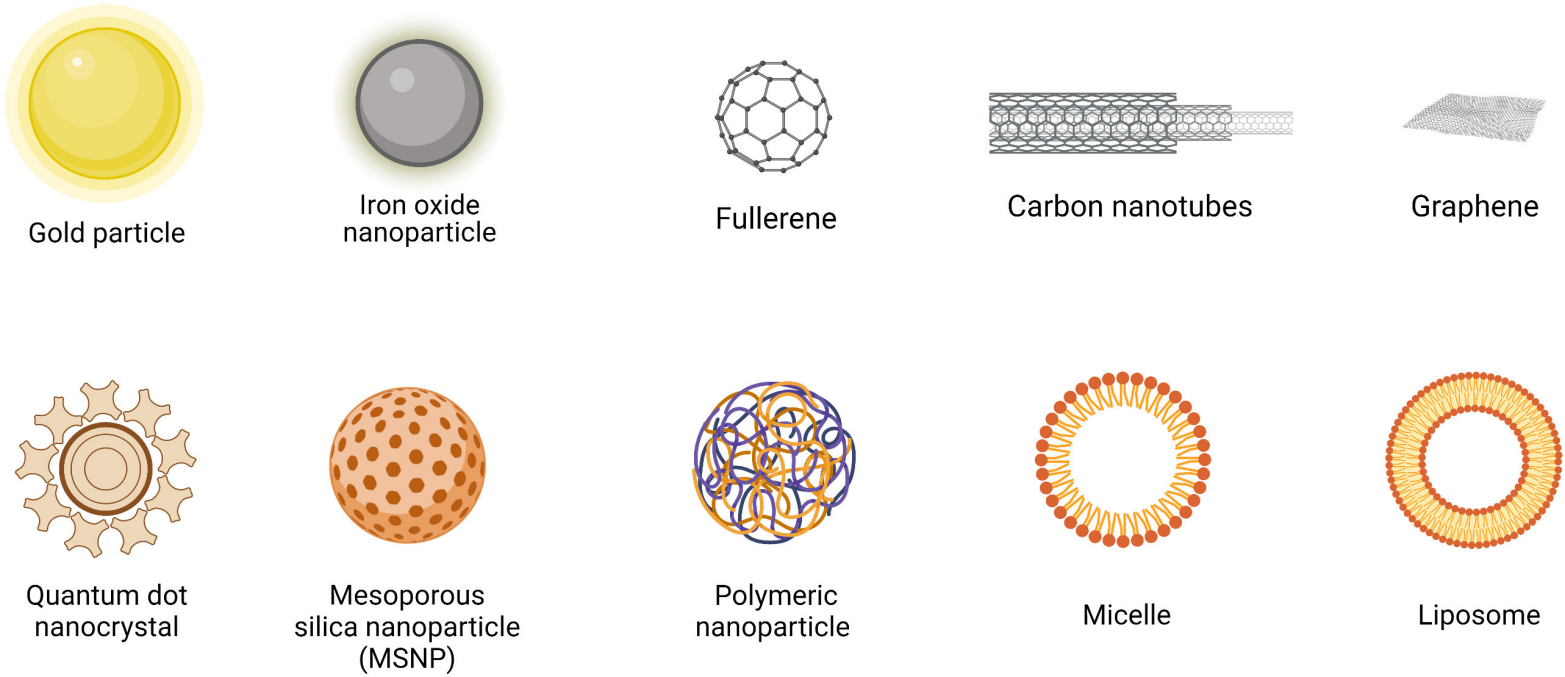
Structures of commonly used nanomaterials (NMLs). Created in BioRender.com.

**Figure 2:**
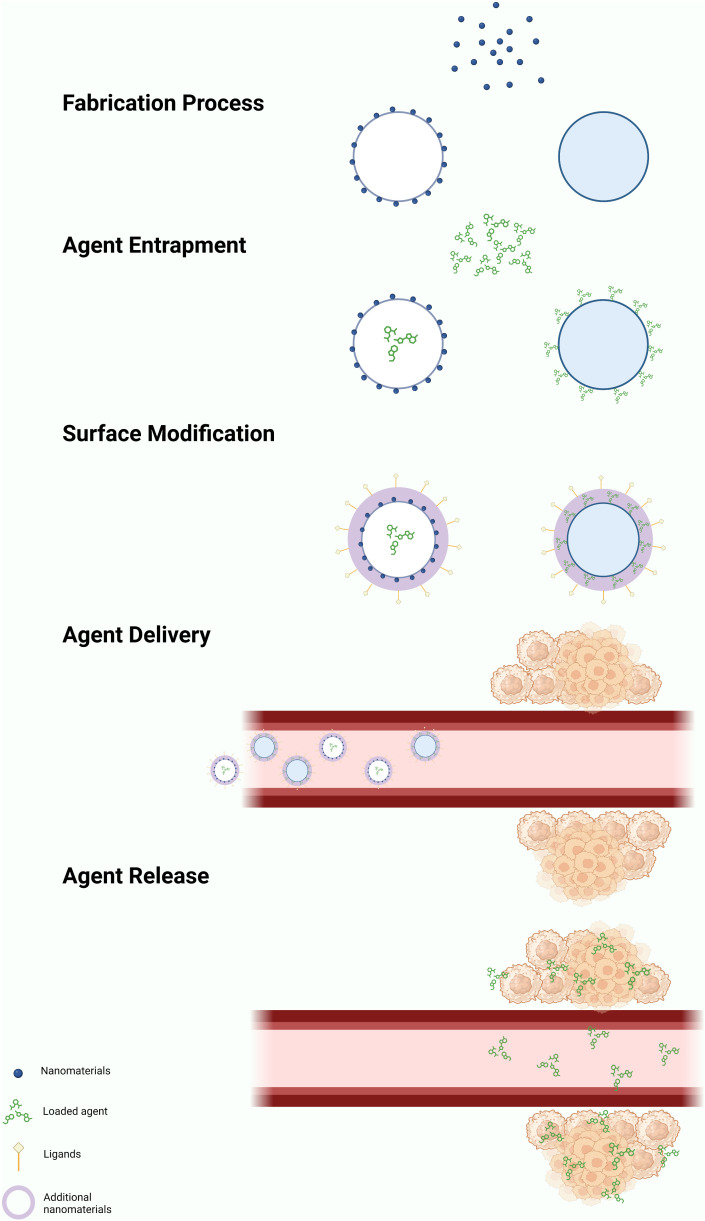
General mechanism of action of nano-delivery system. The formation of NPs can be achieved through either a ‘top-down’ or ‘bottom-up’ approach, resulting in the formation of nanovesicles or nanospheres. Drugs, radiolabel ligands, or light-sensitive agents attached on the NPs through passive or active drug loading mechanisms. Passive drug loading involves encapsulating the drugs during NP formation, while active loading occurs after the NP has formed. The drugs can be encapsulated within the nanovesicles or attached to the surface of the nanospheres. Additional nanomaterial coating is applied to enhance the performance of the nanocarrier. Nanocarriers deliver drugs using both active and passive mechanisms. The enhanced permeability and retention (EPR) effect facilitates passive delivery, leading to the selective accumulation of NPs in the tumor microenvironment (TME). Target ligands aid in delivering the nanocarriers to specific locations. The drugs are released within the TME through various mechanisms, including diffusion, solvent-induced release, chemical reactions, and stimulus-controlled release. Created in BioRender.com.

Engineered nanomaterials (ENMs) is the official terminology used to describe NP with a size of 100 nm or less in at least one dimension and nanostructured materials (32, 33). Based on the compositions, ENMs can be subdivide into organic NMLs, inorganic NMLs, carbon NMLs, metallic NML and polymer based NMLs (34) (**Figure 1**; **Table 1**). The delivery of drugs involves a series of steps. It begins with the formation of the nanostructure, followed by the surface decoration of the nanocarrier (NC), transportation of the NCs, and ultimately, the release of the drug at the target site (**Figure 2**).

**Table 1A:** Overview of commonly used nanomaterials (NMLs)- Main categories of NMLs.

Nanomaterials (NMLs)	Example
Organic NMLs (35)	RNA, micelles, dendrimers and liposomes etc.
Inorganic NMLs (36)	Silicon dioxide (SiO_2_), superparamagnetic iron oxide (SPIO), mesoporous silica NP (MSNP) and quantum dots (QD) etc.
Carbon-based NMLs (37)	Graphene, fullerenes and carbon nanotube (CNT) etc.
Metal-based NMLs (38)	Silver (Ag), gold (Au), copper (Cu), zinc (Zn) and platinum (Pt) etc.
Polymer-based NMLs (39)	Chitosan, polylactic acid (PLA), polyglutamic acid (PGA), poly (lactic-glycolic acid) (PLGA), etc.

**Table 1B:** Overview of different nanomaterials (NMLs)- Detailed features’ demonstrations.

	Liposomes	Dendrimers	Mesoporous silica	Quantum Dots	Gold	Superparamagnetic Iron oxide (SPION)	Polymeric nanoparticles
Common method of fabrication	Thin-film hydration; Ethanol injection; Double emulsion.	Formulation; Nano-construct.	Hydrolysis and condensation	Hydrodermal cutting; Precursor pyrolysis; Hydrothermal aggregation; Reverse micelle synthesis; Microwave-assisted methods; Hydrothermal and solvothermal routes	Colloidal method; Galvanic replacement; Place exchange reaction; Ripening approaches; Citrate reduction of the Au salt.	Coprecipitation and microemulsion; Thermal decomposition; Polyol method; Sono chemical method; Spray and laser pyrolysis; Biomimetic synthesis; Electrochemical deposition; Sol-gel synthesis; Hydrothermal synthesis.	Emulsion/microemulsion polymerization; Interfacial polymerization; Precipitation; Emulsion evaporation, emulsion diffusion, solvent displacement and salting out.
Size	30nm -			2-10 nm	5-40 nm	10-100nm	50-300nm
Shape	Spherical	Bifurcated, mono disperse 3-D globular shape	Mesoporous rods/sphere	Spherical and tetrahedral	Spheres, nanorods, and nano-cages, hollow	Spherical, rhombic, cubic, rod-shape	Spherical, needle, linear, star shape
Types	Unilamellar vesicles (ULVs); Oligolamellar vesicles (OLVs); Multilamellar vesicles (MLVs).	Poly-amidoamine (PAMAM); Poly (propylene imine) (PPI); Poly-L-lysine; Melamine; Poly (etherhydroxylamine) (PEHAM); Poly (esteramine) (PEA); Polyglycerol.	MCM-41; MCM-48; SBA-15; Core-shell MSNs; Hollow MSN.	Core-Type Quantum Dots; Core-Shell Quantum Dots; Alloyed Quantum Dots.	Spherical gold nanoparticles (Au NSs); Gold nanorods (Au NRs).	Magnetite (Fe3O4), maghemite (y-Fe2O3) and mixed ferrites (MFe2O4 where M = Co, Mn, Ni or Zn); Hematite (α-Fe2O3).	PCL(poly(ϵ-caprolactone); PEG (poly(ethylene glycol); PLA (poly(lactic acid); PLGA (poly(lactide-co-glycolide).
Drug entrapment method	Active, Passive; Drug-Lipid conjugation; Combination (active + passive loading).	Simple encapsulation; Electrostatic interaction; Covalent conjugation.	Sol-gel method	PEG conjugation; Passive.	Direct conjugation (ionic/covalent bonding, physical absorption)	Covalent linkage; Electrostatic interactions; Hydrophobic/hydrophilic interactions; Affinity interactions.	Active; Passive.
Surface modifications	PEG coating; Ligands attachment.	Amine-terminated; Ligands attachment.	PEG coating; Ligands attachment	PEG coating; Ligands attachment.	Polyethyleneimine (PEI) coating; PEG coating; Ligands attachment.	PEG; Dextran; Ligands attachment.	Ligands attachment
Pros	Able to deliver hydrophilic and hydrophobic drugs; Extending half-life of the drug; Non-toxic; Biocompatible; Biodegradable.	Able to deliver hydrophilic and hydrophobic drugs; Narrow polydispersity index; Excellent control over molecular structures; Stability; High and fast drug dissolution.	Able to deliver hydrophilic and hydrophobic drugs; High drug loading; Highly porous structure and adsorption capacity Tunable particle size; Easy functionalization; Excellent biocompatibility.	Can be use in theranostic field; Able to deliver hydrophilic and hydrophobic drugs; Smaller size; Larger specific surface area; Higher and more reactivity activity centre; Stronger adsorption capacity; Able to cross blood brain barrier.	Unique optical properties; Ease of synthesis and chemical stability; Biocompatible; Low inherent toxicity; High surface area; Ability to easily functionalize with biomolecules.	Superparamagnetic properties; Nontoxic; Highly biocompatible; Heating properties.	Biodegradability and biocompatibility; Non-toxic.
Delivery of the drug	Passive delivery (EPR); Active delivery (surface modification of the NP).	Passive delivery (EPR); Stimuli-responsive (pH, temperature); Active delivery.	Passive delivery (EPR); Active delivery; Stimuli-responsive (pH, magnetic field, light, temperature).	Passive delivery; Active delivery; Stimuli-responsive (pH, light, heat, radio frequency or magnetic fields).	Stimuli-responsive (magnetic field, light, heat, pH, electroporation); Active delivery.	Passive targeting; Active targeting; Stimuli-responsive (magnetic field).	Passive targeting (EPR); Active targeting; Stimuli-responsive(magnetic).
Administration route	Parenteral; Pulmonary; Oral; Transdermal; Opthalmic; Nasal.	Transdermal; Oral; Ocular; Pulmonary; Targeted drug delivery.	Intravenous; Subcutaneous; Intramuscular; Intratumoral; Ophthalmic; Pulmonary; Nasal; Dermal; Oral.	Intravenous; Oral.		Parenteral; Intervenous; Oral.	Oral; Parenteral; Ocular.
Reference	(40–42)	(41, 43, 44)	(42, 45–47)	(41, 48–51)	(52, 53)	(54–56)	(57, 58)

The construction of the nanostructure can be achieved through two methods, which are ‘top-down’ and ‘bottom-up’ methods (59). The ‘top-down’ approach involves breaking down the bigger particles into smaller pieces using techniques such as interfacial deposition, nanoprecipitation, emulsion, and coacervation. On the other hand, the ‘bottom-up’ method involves building a NML from an individual molecule through processes such as polymerization, molecular inclusion, polycondensation and emulsion (60–62). The NCs are targeted to have a size of ≤150 nm to ensure their ability to enter or exit fenestrated capillaries in the TME while minimizing their perfusion to the kidney, heart, and lung tissue (63–65).

The drugs, contrast or radioactive probe can be loaded onto the surface or entrapped inside the NCs through active or passive drug loading (40). Passive drug loading involves encapsulating the drug during preparation of the NCs, while active drug loading refers to loading the drug after the NCs are formed.

Once the NMLs are formed, the surface of the NMLs can be decorated with receptor-specific ligands to enhance the binding affinity and specificity of biomolecular interactions. For instance, ligands such as ACUPA (66) and PSMA-1 (67) are used to target PSMA^+^ cancer cells. In addition, PEG coating is widely used to modify the surface of the NCs due of its characteristics of high flexibility, enhanced stability and the ability to elongate the vascular circulation of nanoparticles. Consequently, this leads to a reduction in the premature clearance rate of NPs within the vascular circulation (41, 45, 52–54).

The release of the drugs from the NCs can occur in various mechanisms: diffusion, solvent-induced release, chemical reaction and stimuli-controlled release (68, 69). Being a pivotal element in drug delivery, the efficacy of NPs is influenced by a multitude of factors. These encompass the types and attributes of NPs, including shapes, sizes, stability, surface functionalization, physicochemical properties, and more. In a study by Kinnear et al. (70), they have demonstrated the different interactions between the shapes of the NCs and the body system in both *in vivo* and *in vitro*. Their findings revealed that anisotropic particles, which have a prolonged circulation time, perform better in tumor targeting compared to the spherical particles. However, the researchers emphasized that the effect of passive targeting should not be ignored despite of the geometry of the particles considering the complex nature of tumors (**Table 2**).

**Table 2:** Recent developed nanovesicles of PSMA-related drug delivery systems in PCa therapy.

Types of nanovesicle	Designed nanovesicle	Ligands	Target tissue	Function	Author (Reference)	Year
Dendrimers	G4(MP-KEU)		PSMA^+^ PC3 PIP tumors	Radiolabel delivery	Wojceich et al. (71)	2019
Polymeric NP	Folate-HBPE(CT20p)	Folic acid	PSMA^+^ LNCaP cells PSMA^+^ PC3	Peptide delivery	Flores et al. (72)	2017
	ACUPA‐PEG‐PLA‐GBA,	ACUPA	PSMA^+^ LNCaP cells	Drug delivery	Afsharzadeh et al. (73)	2020
	[89Zr]PEG-(DFB)3(ACUPA)1 [89Zr]PEG-(DFB)1(ACUPA)3	ACUPA	PSMA^+^ PC3-Pip	Radiolabel delivery	Meher et al. (74)	2022
	111In/Lu-Nanotexaphyrin	PSMA ligand	PSMA^+^ PC3-PIP PSMA^-^ PC3flu	PDT drug delivery	Cheng et al. (75)	2022
	Chitosan-Coated, Quercetin-Loaded PLGA Nanoparticles	Folic acid	PSMA^+^ PC3-PIP PSMA^-^ PC3flu	Drug delivery	Essa et al. (76)	2023
Liposome	64Cu-DOTA-scFv-Anti-PSMA	Anti-PSMA scFv-cys antibody	PSMA^+^ LNCaP cells	Radiolabel delivery	Wong et al. (77)	2017
	GPL	PSMA antibody	PSMA^+^ LNCaP cells PSMA^+^ PC3	Drug delivery	Tian et al. (78)	2021
Mesoporous Silica	PSMA-Targeted Mesoporous Silica Nanoparticles	anti-FOLH1 monoclonal antibody, clone C803N	PSMA^+^ LNCaP cells		Eva et al. (79)	2019
	223RaA-Silane-PEG-D2B	anti-PSMA D2B antibody	PSMA^+^ LNCaP C4-2 cells	Radioligand delivery	Lankoff et al. (80)	2020
Metallic NP	68Ga-mNP-S1/2 68Ga-mNP-N1/2	Glu-ureido-based PSMA ligand bombesin peptide	PSMA^+^ LNCaP cells GRPR PC3 cells	Radioligand delivery	Liolios et al. (81)	2022
Carbon nanotube	CNT-PTX-PSMA	Anti-PSMA antibody	PSMA^+^ LNCaP cells	Drug delivery	Comparetti et al. (82)	2020
Protein	APODOX	Anti-PSMA antibody	PSMA^+^ LNCaP cells	Drug delivery	Dostalova et al. (83)	2018
Amphiphilic block copolymer	[89Zr]DFB (25) [89Zr]DFB (25)ACUPA (84)	DFB ACUPA	PSMA^+^ PC3-Pip	BNCT therapy	Meher et al. (85)	2021
Cationic nanobubbles (CNB)	siFoxM1-Apt-CNBs	A10-3.2 aptamer	PSMA^+^ LNCaP cells	Gene delivery	Wu et al. (86)	2018

## 3 Nano-theragnostics based on PSMA-targeted molecular imaging

### 3.1 MRI

MRI plays a crucial role in not only the diagnosis but also the treatment of PCa, as it helps to reduce the risk of overdiagnosis and overtreatment by providing the required information in a non-invasive manner (87). For patients with suspicious PCa or at risk for PCa, MRI has already been involved as the preferable imaging tool due to its promising spatial and soft tissue resolution (88–90). While in terms of PCa therapy, the accurate delineation of planned target volume (PTV) and organs at risk (ORA) based on the medical images acquired is a fundamental prerequisite for the successful delivery and positive outcomes of radiotherapy (91–93). The conventional contrast agents used in MRI are transition metallic elements and paramagnetic lanthanide-containing cations such as gadolinium (III) (Gd^3+^) and manganese (II) (Mn^2+^) (94, 95) agents. But the relatively small size of these agents and their poor sensitivity and rapid renal clearance somewhat limit the high resolution-target imaging data acquisition (96, 97). Therefore, a number of NPs were designed using different NMLs as MRI contrast agents to optimize the imaging quality (98–103). However, constructing the multifunctional PSMA-targeted NPs will be more clinically relevant not only as nano-MRI contrast agents but also as nano-delivery vehicles for target delivery of therapeutic medications (104). Barnejee et al. (105) synthesized low-molecular-weight Gd^3+^-based T_1_-weighted contrast agents targeting PSMA to testify their potential for target-specific MRI imaging. By adding one to three Gd^3+^complexes to each designed PSMA-target agents, three desired models engaging Lys–Glu urea as the targeting components for PSMA were then produced. The relaxometric properties of these agents were evaluated in different media including solution, PC3 PIP and PC3 flu PCa cells, and *in vivo* experimental mice models, respectively. Overall, the positive PSMA-mediated contrast enhancement and specificity were indicated suggesting the probability of PSMA-based MRI molecular imaging. In order to further verify the feasibility of PSMA-based NPs for MRI, several *in vivo* studies have been therefore carried out (106–108). Zhu et al. (106) prepared PSMA-targeting-superparamagnetic iron oxide NPs (SPIONs) labelled with polypeptide CQKHHNYLC as MRI contrast agents. PSMA^+^ LNCaP and PSMA-negative (PSMA^-^) PC3 cells were involved in accessing the specificity and *in vivo* MRI were applied to experimental mice to monitor the signal alterations. Significant T_2_ signal reduction from two to twelve hours after injection of the PSMA-targeting-polypeptide-SPIONs were observed with all mice incubated with LNCaP cells compared with unremarkable signal changes in the control group with PC3 tumor-bearing mice. The results exhibited the PSMA-target specificity yet the MRI enhancement efficacy of the designed NPs. Moreover, Tse et al. (107) conjugated a specific anti-PSMA monoclonal antibody named J591 with monocrystalline iron oxide NPs (MNPs) to increase the MRI imaging of PCa. The established LNCaP tumor-bearing mice with J591-MNP or MNP injection were underwent MRI. The obvious contrast enhancement of the experimental group with J591 MNP injection was observed while no prominent changes appeared to the MNP injected-control group showing the pronounced imaging capability of J591 MNPs. Pathohistological examinations were performed on the excised tumors of the mice from both experimental and control groups, and marked iron deposition of the mice from the experimental group compared with little iron accumulation of the mice from the control group were observed, indicating the enhanced target specificity by J591 MNPs. Because of their high clinical expectations, the use of PSMA-targeted NPs as MRI contrast agents is constantly evaluated. Behnam et al. (84) designed a PSMA-targeted bionized nanoferrite (BNF) NP. At low concentrations, they found the PSMA-targeted BNF NP exhibited both the imaging capability and target specificity with diminished NP accumulation in organs of the reticuloendothelial system, thus enhancing the NP concentration in the target regions and thereby improving PSMA^+^ tumor retention and facilitating target images obtaining. Based on their previously conducted PSMA-targeted BNF NP, Ngen et al. (108) used the same NP to evaluate its potential to serve as MRI contrast agents at high concentrations. After applying high-resolution T_2_-weighted MRI *in vivo* to both PC3 PIP-inoculated and PC3 flu-inoculated mice, marked contrast enhancement were shown in the PSMA^+^-tumor-bearing region than the PSMA^-^ one up to 48 hours post-PSMA-targeted BNF NP injection. Meanwhile, the designed NP was shown to be preferentially transported to the peripheral parts of PSMA^+^ and PSMA^-^ tumors. Overall, after the pre-clinical evaluation, PSMA-targeted BNF NPs were possible to be employed for image-guided therapy.

### 3.2 Ultrasound

In contrast to alternative imaging modalities like computed tomography and MRI, ultrasound plays a vital role in the diagnosis and treatment of PCa, considering the nature of rapid and real-time imaging, the absence of radiation, the high availability, and the advantages of economy and portability (109, 110). However, the molecular imaging using ultrasound is lagging far behind due to insufficiency of appropriate nanoscale based-molecular reporters (111, 112). Fan et al. (113) constructed a new kind of lipid nanobubbles (NB) that can specifically bind to PSMA^+^ PCa cell line after modification with anti-PSMA aptamer A10-3.2. The constructed NB were then injected into the experimental mice with the abdominal Doppler monitoring, they found that the ultrasonic imaging ability was significantly enhanced, suggesting the potential of using the designed NB as the PCa-targeted ultrasound contrast agents. As Fan et al. have already demonstrated the diagnostic probability of PCa using A10-3.2 aptamer NB, more recently, Wu et al. (114) developed NB also targeted A10-3.2 aptamer but loaded with Paclitaxel, aiming at testifying the theragnostic potential of the newly synthesized NB named PTX-A10-3.2-PLGA NB. After verification of their target specificity to PSMA, they commenced the injection of PTX-A10-3.2-PLGA NB under low-frequency US triggering. The survival of PSMA^+^ tumor-bearing mice was shown significantly increased, indicating both US imaging and chemotherapeutic ability of the multimodal NB.

Photoacoustic imaging (PAI) stands as an innovative imaging technique that capitalizes on the optical absorption characteristics of diverse chemical components within tumor tissues. This approach yields high-contrast structural imaging, generated from the resultant acoustic waves through a photo-induced ultrasound imaging mechanism (115–117). Given its advantages of non-ionizing radiation, non-invasiveness, deep penetration, and low cost, PAI has shown intriguing potential in facilitating the diagnostic process across diverse disciplines (118, 119). Wang et al. (120) engineered multifunctional NBs (PSMAP/ICG NBs) loaded with indocyanine green (ICG), with a specific targeting affinity for PSMA. These NBs were designed to facilitate ultrasonography, PAI, and fluorescence imaging (FLI) of PCa. The imaging capabilities were assessed in both PSMA^+^ and PSMA^-^ tumor xenografts models. Following a series of validations, the researchers discovered that the performance of ultrasonography and PAI using PSMAP/ICG NB in PSMA^+^ tumor xenografts was drastically elevated compared to the PSMA^-^ tumors, resulting in a noticeable prolongation of both ultrasound and photoacoustic signal intensity enhancements. Additionally, FLI demonstrated a protracted accumulation period of PSMAP/ICG NB in mice bearing PSMA^+^ tumors. Based on these observed results, they concluded that the constructed NB facilitate more intuitive tumor-targeted imaging and could potentially aid in the early identification and diagnosis of PCa. More recently, Kim et al. (121) have designed a new PAI agent by constructing the micelles NP incorporated with PSMA-targeting porphyrin-based molecules (PMP). Their objective is to investigate the utilization of these micelles as theragnostic substances for PCa. The LNCap and PC3 models were engaged to compare the specific PSMA^+^ - binding and targeting capacities. The *in vivo* animal experiment demonstrated a heightened signal intensity compared to the ordinary PAI agent, giving hope for PMP to be one of the possible variants for future PCa theragnosis.

## 4 Applications of PSMA-based nanomedicine in PCa therapy

### 4.1 PSMA-based nanomedicine in chemotherapy

For mCRPC patients, despite lack of curative treatment strategies, chemotherapeutic drugs such as docetaxel and cabazitaxel are recommended. Presently, the primary approach, as per the EAU guidelines, involves their combination with antihormonal drugs, serving as the first-line option (5). Clinical trials have always been conducted to evaluate the efficacy of selected anti-androgen, chemotherapeutic agents and to discover the promising drug combinations (122–125). While certain well-established studies, such as the CHAARTED trial (126) and CARD trial (123), have yielded favorable outcomes, limitations inherent to traditional chemotherapeutic agents remain evident. These drawbacks encompass short circulation duration, limited bioavailability, interference with the rapid proliferation of normal cells, and the emergence of chemotherapy-related side effects. These factors collectively exert a substantial impact on the quality of life of patients (127, 128). Additionally, late chemotherapy is prone to tumor cell resistance, which significantly reduces the efficiency of treatment. Tumor cell resistance emerges as the malignancy and progression of prostate tumor cells intensify, leading to the development of both extrinsic and intrinsic therapy resistance (129). Consequently, this is the juncture where nanomedicine demonstrates its potential in surmounting drug resistance, exerting inhibitory effects on tumor growth. This approach offers a high tumor inhibition rate while minimizing the occurrence of side effects. Through the synergy of chemotherapy and nano-delivery systems, targeted transportation of loaded chemotherapeutic agents and/or antihormonal drugs is achieved. This approach serves to amplify the presence of chemotherapeutic agents at the tumor site and target specific subcellular organelles. Additionally, it contributes to the reversal of resistance mechanisms through single drug therapy. This intricate strategy not only enhances the therapeutic effect but also leads to diminished drug doses and reduced toxic side effects. Ultimately, this culminates in the enhancement of patients’ survival rates (130–132).

Docetaxel, a taxane-based chemotherapeutic agent, finds application in the management of various metastatic and non-resectable tumor types, such as breast cancer, non-small cell lung cancer, PCa, advanced gastric cancer. Its approval in 2004 for the management of mCRPC marks a significant milestone. To this day, it retains its status as a first-line treatment drug for advanced PCa, as stipulated by the EAU guidelines (5, 133). Its mechanism of action had allowed benefits of reducing toxicity, broadening the antitumor spectrum hence prolonging patient survival (134). Meanwhile, it shows the greatest efficiency not only as an individual drug, but also as a component of combinational therapy with ADT or other chemotherapy drugs. On account of the benefits of docetaxel and the extensive advantages of PSMA, Aleksei et al. (122) created an easy way to synthesize docetaxel conjugate and their biological substance in order to construct targeted conjugates based on docetaxel and low molecular weight PSMA ligand. More recently, Fateme et al. (135) have developed a docetaxel-loaded NP of poly (lactic-co-glycolic acid) polyethylene glycol, which fuse to a urea-based anti-PSMA ligand named glutamate-urea-lysine for targeted delivery in PCa. This consequently improve the antitumor efficacy of docetaxel. Besides that, Prashanth et al. (136) had also engineered a variant of novel docetaxel loaded PSMA targeted superparamagnetic iron oxide nanoparticle (SPION) for PCa therapy. Due to its remarkable cellular uptake, accumulation and release of loaded therapeutics in PC cells, it managed to exhibit potent anti-cancer efficacy as well as inhibit chemo-resistance associated protein in PCa cell lines. BIND-014, another variant of PSMA targeted docetaxel loaded biodegradable polymer with prolonged persistence of docetaxel-encapsulated circulation NP formulation, had also been developed and had undergone trials in patients with solid tumors. The results showed that there are no unexpected but only some manageable toxicity, accompanied by a unique pharmacokinetic profile compared with conventional docetaxel (137).

Cabazitaxel, a next-generation taxane, has been approved for the treatment of mCRPC in post-docetaxel patients. Compared to docetaxel, cabazitaxel has a different safety record, with a lower incidence of typical docetaxel-related complications such as alopecia, peripheral neuropathy, peripheral edema, and nail disorders (123, 138). On account of the advantages of cabazitaxel, Cohen et al. (139) developed novel selective PCa-targeting NP containing cabazitaxel to reduce the invasiveness in metastatic PCa cells, which is the disease that symbolized the beginning of the later stage. They designed polyethylene glycolyzed nanostructured lipid carriers decorated with selective ligands targeting PSMA. The NPs uptake initiated via receptor-mediated endocytosis and promote intracellular release of the drug after targeting to PSMA. They concluded that targeted NPs demonstrated effective encapsulation, high specificity and effective eradication selectivity of cabazitaxel on PSMA-positive PCa cells. This outcome contributes to the enhancement of PCa treatment efficacy, achieving dual goals of reducing drug dosage and mitigating adverse toxicities.

### 4.2 PSMA-based nanomedicine in radiation therapy

Radiotherapy is an important component of PCa treatment to localize the tumor, set the radiotherapy plan, and evaluate radiotherapy efficacy under the guidance by a complex series of multimodal medical imaging techniques (140). There is no substitute for radiotherapy, either as preoperative neoadjuvant radiotherapy or as standard postoperative treatment, alongside chemotherapy (141, 142).

#### 4.2.1 Radiosensitization

**Figure 3:**
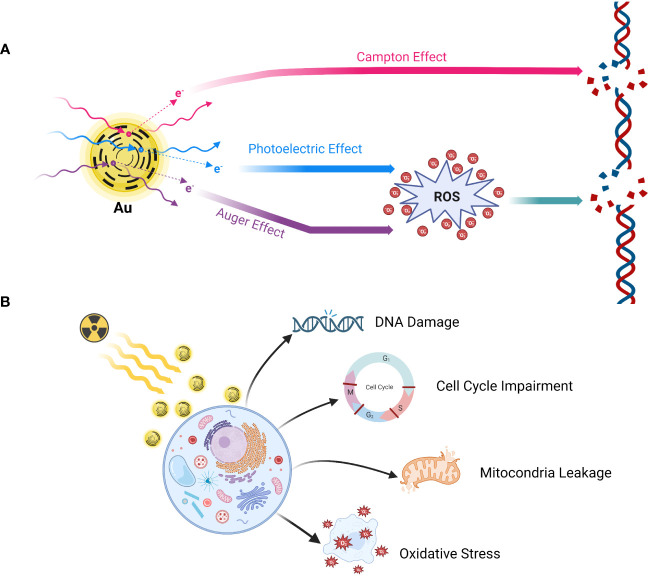
Mechanism of action of gold nanoparticle radio sensitization. **(A)** Physical reaction; **(B)** Biological reaction. Gold nanoparticles (GNPs) extend their anti-tumor function through a combination of physical and biological reactions, leveraging their unique properties. The three main completed physical mechanisms, namely Compton, Photoelectric, and Auger effects, play crucial roles in the tumor-killing effect. DNA damage is achieved through both direct influence from the Compton effect and indirect influence caused by the production of reactive oxygen species (ROS), which follows the Photoelectric and Auger effects. By combining radiotherapy with the administration of GNPs, a series of cellular interactions disrupt numerous biological events, subsequently leading to DNA damage, leakage of mitochondria, and ultimately, cell death. Created in BioRender.com.

To achieve an optimal gain ratio, it is crucial not only to accurately map the PTV and ORA but also to ensure the proper implantation of radiosensitizers. This synergistic approach amplifies the responsiveness of tumor cells to radiation by augmenting the effective dosage delivered to these cells while minimizing impact on normal tissues. This is achieved through the acceleration of DNA damage, ultimately heightening the anti-tumor effect (92, 93, 143, 144). Among high atomic number (Z) metallic NMLs, gold NP (Z=79) has been well-studied based on its physicochemical properties and its induced cellular biological actions (145–147) (**Figure 3**). Gold nanoparticles (AuNP) extend their anti-tumor function through a combination of physical and biological reactions, leveraging their unique properties (144, 147). The three main completed physical mechanisms, namely Compton, Photoelectric, and Auger effects, play crucial roles in the tumor-killing effect (148). General physicians do not require an in-depth understanding of the detailed mechanisms of action. However, it is important to note that DNA damage is achieved through both direct influence from the Compton effect and indirect influence caused by the production of reactive oxygen species (ROS), which follows the Photoelectric and Auger effects (144, 147, 148). By combining radiotherapy with the administration of GNPs, a series of cellular interactions disrupt numerous biological events, subsequently leading to DNA damage, leakage of mitochondria, and ultimately, cell death (144, 147, 148). Luo et al. (149) designed PSMA-targeted ultrasmall gold nanoparticle clusters (CY-PSMA-1-Au_25_ NCs) by combining 1.5 nm Au ions and PSMA-1 ligands as radiosensitizers for PCa radiotherapy. After i.v. administration of the designed NCs into the mice expressed both PSMA^+^ PC3pip cells and PSMA^-^ PC3flu cells, Au content in PC3pip cells were significantly higher than that in PC3flu cells showing the prominent uptake and positive targeting effects. Compared with non-Au NCs targeted-PC3pip cells, PSMA-1-Au25 NCs-incubated PC3pip cells demonstrated pronounced reactive oxygen species (ROS) generation after 6 Gy irradiation showing the marked radio sensitizing effects. Meanwhile, the NCs can be excreted through the kidney very shortly, which effectively reduces the radiation damage to normal tissues during radiotherapy. To further validate the applicable values and effectiveness of NPs in MRI-guided PCa radiotherapy, they constructed new Au-Gd (III)-PSMA NPs by embedding Gd (III) contrast agent onto the surface of PSMA-targeted AuNPs for targeted delivery of Gd (III) to PCa cells. Besides the significant radio sensitizing effects represented by marked tumor growth inhibition against radiotherapy, the results also demonstrated more sensitive MRI imaging efficacies showing the possibility of combining the additional agents with NPs (150).

#### 4.2.2 Radioligand Therapy

Radioligand therapy (RLT), also known as radionuclide therapy, is the use of radiolabeled ligands to emit radiation to destroy tumor cells. In particular, impairment of the structures and biochemical properties of the biological macromolecules after the direct and indirect action of radiation exposure, which further leads to the disturbances and dysregulations of cell reproduction, metabolic function as well as cellular senescence or death, thus achieving the purpose of treatment (151, 152). Several radionuclides including ^125^I (T1/2 = 60.2d) (153), ^131^I (T1/2 = 8.2d) (154), ^188^Re (T1/2 = 16.9h) (155), ^177^Lu (T1/2 = 6.7d) (156) and ^68^Ga (T1/2 = 68min) (157) etc. have already been applied into the clinical practice. Different half-life, irritation energy and the emitted particles make them uniquely useful (158). Currently, PSMA based-^177^Lu RLT engaged either PSMA ligand 617 (159) or PSMA I&T (imaging and therapy) (160) and delivered β particles to the PSMA^+^ cells and the adjacent TME as a novel theragnostic strategy for advanced PCa (161) (**Table 3**). Kratochwil et al. (162) conducted the initial retrospective study using ^177^Lu-PSMA-617 RLT to treat 30 patients with PSMA^+^ mCRPC. Despite lack of systemic stratifications based on the patients’ characteristics and distinct inclusion criteria, considering the castration-resistant natures, the overall results demonstrated the positive antitumor efficacy with tolerable RLT-related toxicities. Moreover, several similar retrospective studies were carried to evaluate the effectiveness and safety of ^177^Lu-PSMA RLT (163–166). Although the results showing the promising outcomes, the efficacy and safety still remain suspicious due to the insufficient sample size and the retrospective natures of the studies. A multicenter retrospective study involving a large cohort of 145 patients from 12 medical centers with mCRPC was conducted by Rahbar et al. (167) using PSA as the primary endpoint to evaluate the biochemical responses. Any PSA decline occupied in sixty-five percent and seventy-two percent patients after the first cycle and second cycle of RLT, respectively. These findings highlight the positive responses to ^177^Lu-PSMA RLT. Notably, this was accompanied by a decrease in hematological adverse events and an overall favorable toxicity profile. These collective outcomes make ^177^Lu-PSMA RLT a promising and rational radiotherapeutic strategy for managing mCRPC. To substantiate the viability of ^77^Lu-PSMA RLT further,^177^Lu-PSMA RLT, Hofman et al. (168) commenced a prospective, single-center, single-arm, phase 2 trial (ACTRN12615000912583) involving 30 patients diagnosed with mCRPC. In this study, the primary outcome measures encompassed PSA levels, imaging responses, post-RLT toxicity, and quality of life (QoL), providing a comprehensive assessment of the efficacy, safety, and impact on QoL resulting from ^177^Lu-PSMA RLT. Fifty-seven percent of patients had a 50% or greater PSA decline and no treatment-related deaths were observed. The post-RLT side effects were mainly mild to moderate and an improvement of the pain severity and interference scores had the clinical value. Moreover, a prospective multicenter phase 3 study (VISION, NCT03511664) was recently initiated by Sartor et al. (161) involving 831 patients who underwent 2:1 randomization. Radiographic progression-free survival (rPFS) and overall survival (OS) were measured as the alternative primary endpoints. Compare with the control group applying standard treatment regimen only, experimental group using ^177^Lu-PSMA RLT combined standard care demonstrated significant enhancement of both rPFS and OS with lower incidence of severe toxicities and no adverse effects on QoL. Despite high clinical viability, due to the non-optimal specificity and short retention time in the tumor tissue, the application of conventional RLT is somewhat limited and therefore should be investigated. The NP-drug delivery system, on the other hand, can enhance the targeting of radioligands, improve the therapeutic effect and reduce the side effects, and thus possesses a wider application prospect (170, 171). With the advanced technology, Czerwińska et al. (172) designed a novel ^223^Ra-anti-PSMA-NaA nanozeolite model recently. After synthesizing the NaA nanozeolite, the radionuclide ^233^ Ra was engaged to labelize the synthesized NaA nanozeolite followed by surface modification using the epoxy silane functionalized polyethylene glycol (silane-PEG). The ^223^Ra- NaA nanozeolite was then conjugated with anti-PSMA monoclonal antibody (D2B) and ^223^RaA-silane-PEG-D2B was then produced. After comprehensive *in vitro* experiments, more than 95% retention follow the trail of 12 days’ validations in both normal saline and human serum showing the high stability of ^223^RaA-silane-PEG-D2. Moreover, the cytotoxicity was also indicated by independent experiments using MTT assay, the result demonstrated the dramatically diminished metabolic activities in both PCa cell lines DU-145 (PSMA^-^) and LNCaP C4-2 (PSMA^+^) after exposure to ^223^RaA-silane-PEG-D2B compared to that treated with non-^223^Ra labeled, bio-conjugated-NaA-silane-PEG-D2^223^. Meanwhile, the sensitivity of LNCaP C4-2 against the ^223^RaA-silane-PEG-D2 was pronouncedly remarkable than that of DU-145 suggesting its marked target selection of PSMA+ cells than PSMA- one. Overall, despite very limited literature resources, NaA nanozeolites are still considered as the ideal nano-carriers and possess the ability to synergistically enhance the specificity and efficacy of RLT and therefore need to be investigated. Given that a number of Lu-PSMA trials have been clinically evaluated, we anticipate that an increasing number of potential NPs will be explored to target PSMA and serve as vehicles to deliver radiopharmaceuticals for future RLT strategies.

**Table 3:** Recent studies of Lu-PSMA RLT for advanced PCa.

Author [Reference]	Year	Sample sizes	Type of study	Max. therapy cycles		Radioactive regimen (GBq)
Kratochwil et al. (162)	2016	30	Retrospective	3		4-6
Ahmadzadehfar et al. (163)	2016	24	Retrospective	2		4.1 – 7.1
Rahbar et al. (164)	2016	82	Retrospective	**-**		5.9 ± 0.5
Rahbar et al. (165)	2016	28	Retrospective	2		5.92 ± 0.04 (1^st^ cycle) 5.86 ± 0.73 (2^nd^ cycle)
Baum et al. (166)	2016	56	Retrospective	2-4		3.6–8.7
Rahbar et al. (167)	2017	145	Retrospective	4		2-8
Hofman et al. (168) (ACTRN12615000912583)	2018	30	Prospective	4		4.4-8.7
Sartor et al. (161) (NCT03511664)	2021	831	Prospective	4-6		7.4
Calais et al. (169) (NCT03042312)	2021	64	Prospective	4		6.0 (n=23) 7.4 (n=41)

### 4.3 PSMA-based nanomedicine in photothermal and photodynamic therapy of PCa

**Figure 4:**
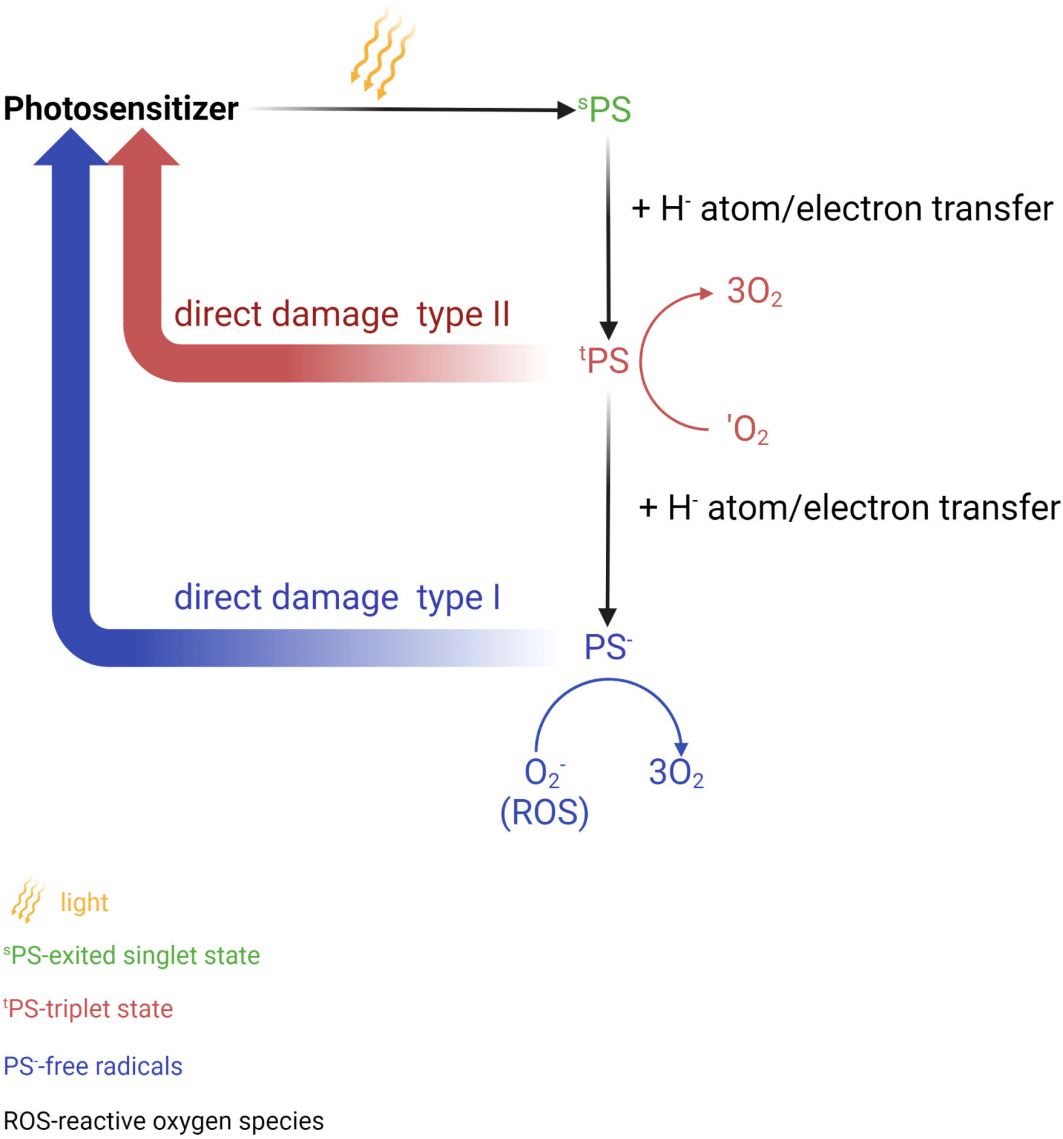
Photochemical reaction in photodynamic therapy (PDT). Photosensitizer (PS) is elevated to the singlet excited state after absorbs a photon of light. This process is followed by the transition to the long triplet excited state after undergoing electron transfer. In this triplet excitable state, the PS can transfer energy to an oxygen molecule, leading to the formation of reactive oxygen species (ROS) (type I) or the highly reactive triplet state (type II). These active oxygen species are responsible for causing damage to biomolecules thus leading to cell death. Created in BioRender.com.

Photodynamic therapy (PDT) and photothermal therapy (PTT) are currently being explored as potential methods of treatment and areas of clinical research these days. PTT utilizes a photothermal agent and a light probe, while PDT relies on a photosensitizer (PS) and a light probe (173–175). PTT kills cancer cells using heat generated through light, while PDT employs various mechanisms such as apoptotic, necrotic, and autophagy-associated cell death (173, 174) (**Figure 4**). Generally, PTT uses a relatively higher light wavelength (800-980nm NIR) compared to PDT, which depends on the peak wavelength of the PS, ranging between 630-730nm (176). (**Table 4**) PDT and PTT employ Monte Carlo models, the standard Pennes bioheat equation, and the first-order thermal-chemical rate equation to calculate the light distributions of the treatment (177–180). Some studies may combine techniques to measure the singlet oxygen level to optimize the PDT treatment plan (181, 182). Up to the present, two PS have been approved for the treatment of PCa. Padeliporfin (WST11, TOOKAD^®^) was clinically approved in 2018 as a PS for patients with previously untreated, unilateral, low-risk prostate cancer. On the other hand, Lu 177 vipivotide tetraxetan (^177^Lu-PSMA-617) was approved in March 2022 for adult patients with PSMA^+^ mCRPC who have undergone androgen receptor pathway inhibition and taxane-based chemotherapy (177).

**Table 4:** Differences between PDT and PTT.

	PDT	PTT
Components	Photosensitizing agent + light probe	Photothermal agent + light probe
Oxygen dependence	Oxygen dependent	Oxygen independent
Type of used light	Diode laser	Near-infrared light (NIR)
Wavelength range	Uses lower wavelength (630-730nm)	Uses higher wavelength (800-980nm)
Nature of the effect	Photochemical	Thermal
Type of cell death	Apoptotic; Necrotic; Autophagy-associated cell death.	Ablation of the tumor cells Thermal sensitization of the tumor cells
Scarring effect	No scarring	May cause scarring

Although the development of PDT and PTT has significantly brought a huge step forward in treating PCa, there are still opportunities for improvement and challenges to address. A few factors that are brought into discussion by researchers include improving the effectiveness of treatment by delivering the PS more effectively (183), prolonging plasma circulation (184, 185), and producing singlet oxygen (186). Luo et al. (187) designed PSMA-targeted cathepsin-activatable AuNP using silicon phthalocyanine (Pc158) that is synthesized and conjugated to AuNP that serve as delivery vehicles through a cathepsin-cleavable linker, GLFGC. AuNPs-Pc158 offers a more precise release as it only releasing in the presence of cathepsin B. However, the authors also acknowledged several factors that might limit the full functionality of the nanomaterials, such as its unavailability to fully penetrate deep tissues, limited NIR light penetration, and reduced effectiveness in a larger tumor due to limited penetration of NIR light into the tumor. Another gold nanocarrier developed by Mangadlao et al. (67) utilizes PSMA-1 as a urea-based ligand and PEG, which are known for their ability to increase the stability, biocompatibility, and circulation half-life of the nanodrug. This PDT nanocarrier, named AuNP-5kPEG-PSMA-1-Pc4 has demonstrated stability in the solvent, which ensures adequate delivery of drugs to the tumor site. It has also been proven by the authors that the nanodrug exhibits high binding avidity, greater selectivity for PSMA-expressing cells, and found no toxicity in animals.

In other experiments, the characteristics of liposomes which can encapsulate hydrophilic and lipophilic drugs have been utilized. Cheng et al. (75) developed a liposome-like NP, named 111In/Lu-Nanotexaphyrin, which exhibits excellent chemical, photo, and colloidal stability. It also effectively generates singlet oxygen, and demonstrates a favorable plasma circulation half-life *in vivo* (T1/2 = 6.6 h). This nanodrug is not only suitable for PDT but also useful for SPECT imaging. Phan et al. (188) have introduced a straightforward one-pot synthetic approach for the production of polydopamine-folate carbon dots (PFCDs) as dual-function theranostic nanocarriers forbioimaging and PTT targeting of PSMA PCa. In the experiment, they utilized polydopamine and folic acid as precursors. PSMA receptors are recognized by folic acid residue, while polydopamine-derived carbon dots will act as the thermal conductor, leading to cell apoptosis. PFCD is easily synthesized via the one-step hydrothermal method. As a contrast, the researchers compared the treatment of different cells using PFCDs: lung cancer cell A549 (which doesn’t express PSMA), normal prostate cell RPWE-1 (with low PSMA expression), and PSMA+ LNCaP PCa cell. The investigation concluded that PFCD exhibited good biocompatibility, negligible cytotoxicity to PCa cells, and effectively targeted PCa cells.

A multifunctional nanocarrier, Ce6@PDA-DCL-PFP, was developed by Dai et al. (189) for ultrasound-guided combined photodynamic/photothermal therapy (PDT/PTT) of PCa. This melanin-like polydopamine (PDA) nanocarrier was bound with a small-molecule PSMA inhibitor, named DCL, and loaded with perfluoropentane (PFP) and the photosensitizer chlorin e6 (Ce6). The researchers compared it with a corresponding non-targeted probe (Ce6@PDA-PEG-PFP) and found out that DCL-PFP provides more advantages, such as higher cellular uptake (6.5 times) and apoptotic effect (92.57%) compared to non-targeted PEG-PFP (35.33%). Both DCL-PFP and PEG-PFP demonstrated stability in phosphate buffered saline (PBS) and cell culture medium. No obvious tissue damage or toxicity was observed after treatment. After all, Ce6@PDA-DCL-PFP has exhibited greater therapeutic effects than Ce6@PDA-PEG-PFP with the presence of DCL. Lei et al. (190) proposed and investigated the use of a biocompatible melanin nanoprobe (PMNs-II-813) in combination with a PSMA antigen small molecule inhibitor. They developed a second-generation ultrafine melanin nanoparticle, MNP (MN-II) combined with ^89^Zr, ^131^I, and Mn^2+^ ions. The study demonstrated that PMNs-II-813 is a stable, biocompatible, and safe nanocarrier. It can be used for long-term multimodal imaging with dual-enhanced PET/MRI as well as radioisotope therapy (RIT). By all means, PMNs-II-813 act as a PTT nanocarrier, while also enabling PET/MRI monitoring and RIT. In a recent study by Xiao et al. (191), FDA-approved poly-lactic-glycolic changeable liquid PFP, photosensitizer IR780, and therapeutic drugs Paclitaxel, to perform PTT combined chemotherapy on PSMA^+^ C4-2 PCa tissues. By modifying the surface of nanocarriers with anti-PSMA antibodies, this P-PIP nanoparticle achieved more precise cell targeting. In addition, the chemotherapeutic drug paclitaxel will only be released under the trigger of NIR light. With the help of NIR light, PFP will be converted from liquid into gaseous microbubbles. The biosafety evaluation of this nanodrug has shown no toxicity compared with the control group based on histopathological changes (**Table 5**).

**Table 5:** Preclinical studies of nanoparticle-based PDT and PTT on PSMA-positive PCa.

Treatment type	Designed nanoparticle	Light dose	Cell line	Year	Author (Reference)
PDT	AuNPs-Pc158	670nm	PSMA^+^ PC3-PIP and PSMA^-^ PC3-flu	2020	Luo et al. (187)
PDT	AuNP-5kPEG-PSMA-1-Pc4	672nm	PSMA^+^ PC3-PIP and PSMA^-^ PC3-flu	2018	Mangadlao et al. (67)
PDT	111In/Lu-nanotexaphyrin	775nm	PSMA^+^ PC3-PIP and PSMA^-^ PC3-flu	2022	Cheng et al. (75)
PTT	PFCD	808nm	PSMA^+^ LNCaP	2019	Phan et al. (188)
PDT/ PTT	Ce6@PDA-PEG-PFP Ce6@PDA-DCL-PFP	660nm 808nm	PSMA^+^ LNCaP PSMA^-^ PC3	2021	Dai et al. (189)
PTT/ RIT	PMNs-II-813	808nm	PSMA^+^ LNCap	2021	Xia et al. (190)
PTT/ Chemotherapy	P-PIP NPs	808nm	PSMA^+^ C4-2	2023	Xiao et al. (191)

### 4.4 PSMA-based nanomedicine in immunotherapy therapy of PCa

Immunotherapy represents a significant branch of treatment, particularly for mCRPC, where conventional therapies have often failed (192). Its unique ability to stimulate the patient’s own antitumor immune response facilitates the elimination of cancer cells (193). In contemporary medicine, immunotherapy offers a patient-tailored approach that can be customized to achieve optimal outcomes, often in combination with chemotherapy, such as conventional cytotoxic agents or androgen receptor therapy (194). PCa is a perfect model for oncological immunotherapy because the prostate is non-essential and has numerous TAAs as potential targets (195). In addition, PCa is a slowly progressive disease that provides sufficient time to generate an anti-tumor immune response (196). Immune checkpoint inhibitors (ICIs) and adoptive cellular immunotherapies (ACT) have garnered significant attention among the various categories of immunotherapy (196). ICIs function by blocking checkpoint proteins (e.g., CTLA-4, PD-L1, and PD-1) from binding to their corresponding partner proteins, enabling T cells to effectively target cancer cells (197–199). On the other hand, ACT, including chimeric antigen receptor-engineered T-cells (CAR-T), bispecific engagers (BiTEs), and Sipuleucel-T, involves the administration of immunogenic cells to elicit an anti-tumorigenic response (197–199).

Despite the range of therapies available today, solid tumors present inherent limitations that impede the effectiveness of immunotherapy, unlike hematological malignancies. These limitations include tumor heterogeneity and the TME (200–202). However, nanotechnology holds promise in overcoming these obstacles in PCa (203). NP have demonstrated the ability to overcome physical barriers in PCa cells, facilitating the access of immune effector cells to cancer cells and enhancing the potential of immunotherapy (204). Furthermore, the combination of nanotechnology with immunotherapy offers the potential to enhance efficacy while reducing the required dosage and mitigating dose-limiting toxicities (205, 206).

The second generation of nanobody-based chimeric antigen receptor (NBPII-CAR), which targets PSMA, was developed by Hassani et al. (202). In contrast to the first generation of CARs, which exhibited limitations such as low cytokine secretion, short lifespan, and poor proliferative capacity, the second generation of CARs demonstrates promising outcomes for clinical applications. In this study, electroporation was employed as a time-saving method to introduce DNA into Jurkat cells, as opposed to the gamma-retroviral/lentiviral system. Following molecular design and codon optimization, NBPII-CAR was commercially synthesized and subcloned in pcDNA3.1(+). Subsequently, the transferred cells were monitored three days after electroporation, with the selective antibiotic Geneticin used to enhance NBPII-CAR expression in Jurkat cells. Subsequent to this, Jurkat cells were co-cultured with the commonly utilized LNCaP and DU145 cell lines in PCa studies. These co-cultured cells were then incubated with PCa cells at various ratios, enabling the assessment of Interleukin (IL)-2 production, CD25 expression, and proliferation. The engineered T-cells were activated by the nanobody-based CAR upon encountering the PSMA-expressing target cells, as examined in this study. Consequently, this activation led to an elevated expression level of CD25, mirroring the trend observed in IL-2 production. Moreover, the proliferation capacity of the engineered T-cells demonstrated a higher rate compared to Jurkat cells subjected to mock-electroporation. Thus, the second generation of nanobody-based CAR exhibits promising results in its ability to recognize PSMA on PCa cell lines, underscoring its potential for targeted immunotherapy.

IL plays a crucial role in the TME by promoting cancer cell survival and proliferation under androgen-deprived conditions (207). The integration of specific IL antibodies has emerged as a promising approach in recent anti-tumor therapy. Recent studies investigating IL-specific monoclonal antibodies (mAb) have demonstrated their potential to enhance the pharmacodynamics of drug-drug interactions without exhibiting toxicities against anti-PSMA or reactivities against anti-CAR agents (208, 209). Multiple researches have been conducted to explore the efficacy of this strategy.

In a study conducted by Wang D et al. (210), the researchers focused on IL-23, a heterodimeric cytokine composed of IL12B (IL-12p4O) and IL23A (IL-23P19) subunits, which plays a vital role in the TME by promoting cell proliferation and survival. To investigate the therapeutic potential of IL-23 antibodies, four panels of CARs were designed and evaluated using a mouse model. These panels included IL23mAb-PSMA-CARs, PSMA-CAR, IL23mAb-T2A-PSMA-CAR, and PSMA-CAR with soluble IL23mAb. Notably, IL23mAb-T2A-PSMA-CAR demonstrated the most promising results, effectively eradicating tumors starting from day 14 post-injection and leading to immediate weight regain in the NOD/SCID IL-2 gamma (NSG) mice model. Furthermore, IL23mAb-T2A-PSMA-CAR exhibited the highest secretion of cytokines, and there was a significant increase in the populations of immune cells such as CD45RO+ CD8+ T cells and CD127+ CD4+ CAR T cells. RNA sequencing analysis revealed distinct gene expression patterns in the mice, which were subsequently confirmed through reverse infusion experiments in the same model. In another study conducted by Sugimoto Y et al. (211), the integration of IL-2 in antibodies-dependent cellular toxicity activity against PSMA was investigated in the presence of human peripheral blood mononuclear cells (PBMCs). Unlike IL-23, IL-2 is a cytotoxic cytokine that promotes tumor killing. The researchers genetically modified the first generation of the mouse-human chimeric IgG1 antibody, known as 2C9 (KM2777), by fusing human IL-2 to its C-terminus. This modification resulted in a new antibody referred to as KM2812. Importantly, KM2812 retained its binding ability to PSMA and exhibited enhanced cytotoxicity against PSMA compared to KM2777. Moreover, KM2812 demonstrated remarkable anti-tumor activity and led to complete regression of tumors in certain mouse models, distinguishing it from the effects observed with KM2777.

An emerging player in this field is extracellular vehicles (EVs). These naturally occurring, engineered modified plasma membrane-bound vesicles serve as couriers for transporting proteins, lipids, and genetic materials (212, 213). They possess the remarkable ability to traverse biological barriers and reach target cells, making them invaluable in the field of nanomedicine delivery (200). Currently, clinical trials are underway exploring the utilization of EVs as carriers for CAR-T therapies (CAR-T EV) (200).

Unfortunately, based on current clinical trials, the results of combining immunotherapy with nano delivery systems do not demonstrate the same level of promise as chemotherapy in the current treatment landscape. However, in the context of advanced PCa, immunotherapy primarily plays a role in slowing disease progression and alleviating adverse symptoms (204). PCa is a typical epithelial adenocarcinoma; therefore, insights for developing immunotherapeutic approaches for PCa also apply to other types of epithelial cancer.

## 5 Conclusion

In the era of precision therapy and personalized medicine, the utilization of a single modality treatment appears to be a practice belonging to the past century. Instead, multimodality combination treatment strategies have emerged as a recommended approach for managing various urological malignancies. With the advancements in biotechnology and ongoing research in nanomedicine, the potential of nano-drug delivery systems to achieve optimal treatment efficacy has become evident (204, 214). The advantages of low immunity and high target specificity make NML a promising biomaterial (215).The application of nanomedicine could not only drastically enhance the sensitivity and specificity of early diagnosis of PCa but also allow for the modification of biochemical and biophysical properties on demand, thereby increasing the drug enrichment and facilitating the drug penetration while diminishing the systemic toxicity of cytotoxic agents, with the ultimate goal of enhancing the treatment efficiency for advanced PCa synergistically (216, 217). Since the successful implementation of Lu-PSMA, PSMA has garnered significant attention, advocating for the use of novel NP that target PSMA due to their high clinical value. As we discussed above, nano-drug delivery systems targeting PSMA play a crucial role in exploring of novel treatment strategies for PCa management. Despite the discrepancies of NML and targeted PSMA ligands, the effectiveness of using PSMA-targeted nano-drug delivery has demonstrated significant priority compared to the conventional drug delivery method, according to vast studies. We have been delivering concepts, applicable NMLs, and outstanding preclinical study outcomes towards the treatment of PSMA-positive PCa through this review. Considering the usage of NPs is still relatively new compared to other forms of treatment that have been practiced for decades and years, there aren’t many approved prostate cancer nanotheranostics materials available on the clinical practice. There are plenty of obstacles to be overcome, such as the necessity to fully understand the toxicity of NPs to the human body, the difficulties of obtaining regulatory approval, ethical concerns, particularly for those NMLs that will need to change the DNA sequence, and the inadequate knowledge of humans toward NMLs. However, the graph’s showing a boost in quantity of research in nanomedicine suggests that a deeper understanding of this particular topic is highly anticipated. A recent algorithm developed by Nikolai et al. (218) has shown promising results in predicting NML cellular toxicity with a remarkably low root mean square error (RMSE) rate of 12% indicating the potential for significant advancements in the near future. Acknowledging that PSMA functions as a TAA rather than a tumor-specific antigen, the deficiency in robust target specificity stands out as a significant concern and hurdle in the development of PSMA-targeted nanodrug delivery systems. Presently, the primary clinical applications revolving around PSMA encompass PSMA-PET CT and radiotherapy (219, 220). Nonetheless, in comparison to other TAAs associated with PCa, such as PSA, prostate stem cell antigen (PSCA), and prostate acid phosphatase (PAP) (221), PSMA exhibits an elevated expression level in PCa cells. This expression surge is further accentuated by the GS and tumor malignancy, particularly in the context of CRPC. These factors collectively endow PSMA with a compelling potential that warrants continuous exploration. While the fundamental biochemical components of NML were briefly introduced, an exhaustive understanding of the intricate mechanisms behind each individual element is not imperative for general physicians. Nevertheless, it is prudent to underscore the significance of a meticulous examination of the specific clinical applications. Such scrutiny holds the potential to influence the of managing advanced solid tumors, including PCa. Yet, the limitations associated with PSMA underscore the necessity for a thorough evaluation and further assessment of its clinical merits. Despite being a non-essential organ, the prostate gland exhibits immunocompetent characteristics (222). Moreover, it goes beyond its primary function by producing valuable chemical compounds like PSMA, which not only find relevance in prostate health but also play a pivotal role in conditions such as rectal and breast cancer. As a result, insights gained from studying PSMA based nano-delivery system could potentially shed light on treatment strategies applicable to a broader range of solid tumors. However, the current studies still remain at the preclinical stage due to the insufficient evidence of the precise mechanism of action and a lack of pharmacotoxicity record, as well as the long-term outcomes. Further investigation should meticulously consider the optimal combination of NML and PSMA ligands to achieve maximum safety and efficacy profiles. It is strongly believed that a novel horizon encompassing the PSMA-targeted nanomedicine-based multimodality combination therapy could bring the Gospel to patients with advanced PCa. The combined PSMA-targeted nanomedicine-based immunotherapy +chemotherapy + radiotherapy strategy will also bring unlimited possibilities for mCRPC, and better multimodality treatments will emerge.

## Author contributions

MZH and YC performed the literature search regarding available databases and drafted the manuscript. JZ and CE helped in reviewing the relevant literature. GDY and XIY assisted in preparing figures. KD evaluated and reinforced the technical background. CKXC and NTZV revised the manuscript. KBH and ME contributed to editing the manuscript. All authors have read and agreed to the published version of the manuscript.

## References

[1] Sung H Ferlay J Siegel RL Laversanne M Soerjomataram I Jemal A. Global cancer statistics 2020: GLOBOCAN estimates of incidence and mortality worldwide for 36 cancers in 185 countries. CA: Cancer J Clin (2021) 71(3):209–49. doi: 10.3322/caac.21660 33538338

[2] He M Cao Y Chi C Yang X Ramin R Wang S. Research progress on deep learning in magnetic resonance imaging-based diagnosis and treatment of prostate cancer: a review on the current status and perspectives. Front Oncol (2023) 13:1189370. doi: 10.3389/fonc.2023.1189370 37546423 PMC10400334

[3] Bruinsma SM Roobol MJ Carroll PR Klotz L Pickles T Moore CM. Expert consensus document: Semantics in active surveillance for men with localized prostate cancer - results of a modified Delphi consensus procedure. Nat Rev Urology. (2017) 14(5):312–22. doi: 10.1038/nrurol.2017.26 28290462

[4] Wilt TJ Ullman KE Linskens EJ MacDonald R Brasure M Ester E. Therapies for clinically localized prostate cancer: A comparative effectiveness review. J urology. (2021) 205(4):967–76. doi: 10.1097/ju.0000000000001578 33350857

[5] Cornford P van den Bergh RCN Briers E Van den Broeck T Cumberbatch MG De Santis M. EAU-EANM-ESTRO-ESUR-SIOG guidelines on prostate cancer. Part II-2020 update: treatment of relapsing and metastatic prostate cancer. Eur urology. (2021) 79(2):263–82. doi: 10.1016/j.eururo.2020.09.046 33039206

[6] Teo MY Rathkopf DE Kantoff P. Treatment of advanced prostate cancer. Annu Rev Med (2019) 70:479–99. doi: 10.1146/annurev-med-051517-011947 PMC6441973 30691365

[7] Antonov P Raycheva G Popov V. Unexpected long-term survival in an adult patient with metastatic prostate cancer. Urol Case Rep (2021) 37:101634. doi: 10.1016/j.eucr.2021.101634 33747795 PMC7973130

[8] Gheorghe GS Hodorogea AS Ciobanu A Nanea IT Gheorghe ACD. Androgen deprivation therapy, hypogonadism and cardiovascular toxicity in men with advanced prostate cancer. Curr Oncol (Toronto Ont). (2021) 28(5):3331–46. doi: 10.3390/curroncol28050289 PMC8482210 34590590

[9] Kirby M Hirst C Crawford ED. Characterising the castration-resistant prostate cancer population: a systematic review. Int J Clin practice. (2011) 65(11):1180–92. doi: 10.1111/j.1742-1241.2011.02799.x 21995694

[10] Meng F Zhu S Zhao J Vados L Wang L Zhao Y. Stroke related to androgen deprivation therapy for prostate cancer: a meta-analysis and systematic review. BMC cancer. (2016) 3 16:180. doi: 10.1186/s12885-016-2221-5 PMC4778362 26940836

[11] Heidenreich A Bastian PJ Bellmunt J Bolla M Joniau S van der Kwast T. EAU guidelines on prostate cancer. part 1: screening, diagnosis, and local treatment with curative intent-update 2013. Eur urology. (2014) 65(1):124–37. doi: 10.1016/j.eururo.2013.09.046 24207135

[12] Andela CD Matte R Jazet IM Zonneveld WC Schoones JW Meinders AE. Effect of androgen deprivation therapy on cognitive functioning in men with prostate cancer: A systematic review. Int J urology: Off J Japanese Urological Assoc (2021) 28(8):786–98. doi: 10.1111/iju.14596 PMC9545697 34128263

[13] Cascone T Leung CH Weissferdt A Pataer A Carter BW Godoy MCB. Neoadjuvant chemotherapy plus nivolumab with or without ipilimumab in operable non-small cell lung cancer: the phase 2 platform NEOSTAR trial. Nat Med (2023) 29(3):593–604. doi: 10.1038/s41591-022-02189-0 PMC10033402 36928818

[14] Kantoff PW Higano CS Shore ND Berger ER Small EJ Penson DF. Sipuleucel-T immunotherapy for castration-resistant prostate cancer. New Engl J Med (2010) 9 363(5):411–22. doi: 10.1056/NEJMoa1001294 20818862

[15] Kellokumpu-Lehtinen PL Harmenberg U Joensuu T McDermott R Hervonen P Ginman C. 2-Weekly versus 3-weekly docetaxel to treat castration-resistant advanced prostate cancer: a randomised, phase 3 trial. Lancet Oncol (2013) 14(2):117–24. doi: 10.1016/s1470-2045(12)70537-5 23294853

[16] Rathkopf DE Smith MR de Bono JS Logothetis CJ Shore ND de Souza P. Updated interim efficacy analysis and long-term safety of abiraterone acetate in metastatic castration-resistant prostate cancer patients without prior chemotherapy (COU-AA-302). Eur urology. (2014) 66(5):815–25. doi: 10.1016/j.eururo.2014.02.056 PMC4418928 24647231

[17] Sweeney C Bracarda S Sternberg CN Chi KN Olmos D Sandhu S. Ipatasertib plus abiraterone and prednisolone in metastatic castration-resistant prostate cancer (IPATential150): a multicentre, randomised, double-blind, phase 3 trial. Lancet (London England). (2021) 398(10295):131–42. doi: 10.1016/s0140-6736(21)00580-8 34246347

[18] Holm M Doveson S Lindqvist O Wennman-Larsen A Fransson P. Quality of life in men with metastatic prostate cancer in their final years before death – a retrospective analysis of prospective data. BMC Palliative Care (2018) 17(1):126. doi: 10.1186/s12904-018-0381-6 30509249 PMC6278096

[19] Tucci M Leone G Buttigliero C Zichi C RF DIS Pignataro D. Hormonal treatment and quality of life of prostate cancer patients: new evidence. Minerva urologica e nefrologica = Ital J Urol nephrology. (2018) 70(2):144–51. doi: 10.23736/s0393-2249.17.03066-1 29241313

[20] Fu S Li G Zang W Zhou X Shi K Zhai Y. Pure drug nano-assemblies: A facile carrier-free nanoplatform for efficient cancer therapy. Acta Pharm Sin B (2022) 12(1):92–106. doi: 10.1016/j.apsb.2021.08.012 35127374 PMC8799886

[21] Shi J Kantoff PW Wooster R Farokhzad OC. Cancer nanomedicine: progress, challenges and opportunities. Nat Rev Cancer. (2017) 17(1):20–37. doi: 10.1038/nrc.2016.108 27834398 PMC5575742

[22] Yang F He Q Dai X Zhang X Song D. The potential role of nanomedicine in the treatment of breast cancer to overcome the obstacles of current therapies. Front Pharmacol (2023) 14:1143102. doi: 10.3389/fphar.2023.1143102 36909177 PMC9992554

[23] Maeda H Wu J Sawa T Matsumura Y Hori K. Tumor vascular permeability and the EPR effect in macromolecular therapeutics: a review. J Controlled release: Off J Controlled Release Soc (2000) 65(1-2):271–84. doi: 10.1016/s0168-3659(99)00248-5 10699287

[24] Nakamura Y Mochida A Choyke PL Kobayashi H. Nanodrug delivery: is the enhanced permeability and retention effect sufficient for curing cancer? Bioconjugate Chem (2016) 27(10):2225–38. doi: 10.1021/acs.bioconjchem.6b00437 PMC7397928 27547843

[25] Evans JC Malhotra M Cryan JF O’Driscoll CM. The therapeutic and diagnostic potential of the prostate specific membrane antigen/glutamate carboxypeptidase II (PSMA/GCPII) in cancer and neurological disease. Br J Pharmacol (2016) 173(21):3041–79. doi: 10.1111/bph.13576 PMC5056232 27526115

[26] Jeitner TM Babich JW Kelly JM. Advances in PSMA theranostics. Transl Oncol (2022) 22:101450. doi: 10.1016/j.tranon.2022.101450 35597190 PMC9123266

[27] Haberkorn U Eder M Kopka K Babich JW Eisenhut M. New strategies in prostate cancer: prostate-specific membrane antigen (PSMA) ligands for diagnosis and therapy. Clin Cancer research: an Off J Am Assoc Cancer Res (2016) 22(1):9–15. doi: 10.1158/1078-0432.Ccr-15-0820 26728408

[28] Donin NM Reiter RE. Why targeting PSMA is a game changer in the management of prostate cancer. J Nucl medicine: Off publication Soc Nucl Med (2018) 59(2):177–82. doi: 10.2967/jnumed.117.191874 PMC6910619 28986509

[29] Sheehan B Neeb A Buroni L Paschalis A Riisnaes R Gurel B. Prostate-specific membrane antigen expression and response to DNA damaging agents in prostate cancer. Clin Cancer research: an Off J Am Assoc Cancer Res (2022) 28(14):3104–15. doi: 10.1158/1078-0432.Ccr-21-4531 PMC9365343 35552383

[30] Hernández-Jiménez T Cruz-Nova P Ancira-Cortez A Gibbens-Bandala B Lara-Almazán N Ocampo-García B. Toxicity assessment of [(177)Lu]Lu-iFAP/iPSMA nanoparticles prepared under GMP-compliant radiopharmaceutical processes. Nanomaterials (Basel) (2022) 12(23):4181. doi: 10.3390/nano12234181 36500804 PMC9739705

[31] Afshar-Oromieh A Avtzi E Giesel FL Holland-Letz T Linhart HG Eder M. The diagnostic value of PET/CT imaging with the (68)Ga-labelled PSMA ligand HBED-CC in the diagnosis of recurrent prostate cancer. Eur J Nucl Med Mol imaging. (2015) 42(2):197–209. doi: 10.1007/s00259-014-2949-6 25411132 PMC4315487

[32] Ramakrishnan S Mahawer SK Prasad M Chaudhary M Kumar A Govindasamy P. Chapter 21 - Nanomaterials in integrated methods for soil remediation (biological/physiological combination processes). In: Amrane A Mohan D Nguyen TA Assadi AA Yasin G, editors. Nanomaterials for soil remediation. Elsevier (2021). p. 445–62.

[33] Albalawi F Hussein MZ Fakurazi S Masarudin MJ. Engineered nanomaterials: the challenges and opportunities for nanomedicines. Int J nanomedicine. (2021) 16:161–84. doi: 10.2147/ijn.S288236 PMC7802788 33447033

[34] Pelaz B Alexiou C Alvarez-Puebla RA Alves F Andrews AM Ashraf S. Diverse applications of nanomedicine. ACS Nano (2017) 11(3):2313–81. doi: 10.1021/acsnano.6b06040 PMC5371978 28290206

[35] Ijaz I Gilani E Nazir A Bukhari A. Detail review on chemical, physical and green synthesis, classification, characterizations and applications of nanoparticles. Green Chem Lett Rev (2020) 13(3):223–45. doi: 10.1080/17518253.2020.1802517

[36] Wang J Wu X Shen P Wang J Shen Y Shen Y. Applications of inorganic nanomaterials in photothermal therapy based on combinational cancer treatment. Int J nanomedicine. (2020) 15:1903–14. doi: 10.2147/ijn.S239751 PMC7094149 32256067

[37] Siqueira JR Oliveira ON. 9 - carbon-based nanomaterials. In: Da Róz AL Ferreira M de Lima Leite F Oliveira ON, editors. Nanostructures. William Andrew Publishing (2017). p. 233–49.

[38] Yaqoob AA Ahmad H Parveen T Ahmad A Oves M Ismail IMI. Recent advances in metal decorated nanomaterials and their various biological applications: A review. Front Chem (2020) 8:341. doi: 10.3389/fchem.2020.00341 32509720 PMC7248377

[39] Han J Zhao D Li D Wang X Jin Z Zhao K. Polymer-based nanomaterials and applications for vaccines and drugs. Polymers (Basel) (2018) 10(1):31. doi: 10.3390/polym10010031 30966075 PMC6415012

[40] Liu P Chen G Zhang J. A review of liposomes as a drug delivery system: current status of approved products, regulatory environments, and future perspectives. Molecules (2022) 27(4):1372. doi: 10.3390/molecules27041372 35209162 PMC8879473

[41] Patra JK Das G Fraceto LF Campos EVR MdP R-T LS A-T. Nano based drug delivery systems: recent developments and future prospects. J nanobiotechnology (2018) 16(1):71. doi: 10.1186/s12951-018-0392-8 30231877 PMC6145203

[42] Lu H Wang J Wang T Zhong J Bao Y Hao H. Recent progress on nanostructures for drug delivery applications. J Nanomaterials (2016) 2016:5762431. doi: 10.1155/2016/5762431

[43] Chauhan AS. Dendrimers for drug delivery. Molecules (2018) 23(4):938. doi: 10.3390/molecules23040938 29670005 PMC6017392

[44] Zhu J Shi X. Dendrimer-based nanodevices for targeted drug delivery applications [10.1039/C3TB20724B]. J Materials Chem B (2013) 1(34):4199–211. doi: 10.1039/C3TB20724B 32261015

[45] Manzano M Vallet-Regí M. Mesoporous silica nanoparticles for drug delivery. Adv Funct Mater (2020) 30(2):1902634. doi: 10.1002/adfm.201902634

[46] Kaushik N Borkar SB Nandanwar SK Panda PK Choi EH Kaushik NK. Nanocarrier cancer therapeutics with functional stimuli-responsive mechanisms. J Nanobiotechnol (2022) 20(1):152. doi: 10.1186/s12951-022-01364-2 PMC8944113 35331246

[47] Sábio R Meneguin A Martins dos Santos A Monteiro A Chorilli M. Exploiting mesoporous silica nanoparticles as versatile drug carriers for several routes of administration. Microporous Mesoporous Materials (2021) 312:110774. doi: 10.1016/j.micromeso.2020.110774

[48] Pawar V Maske P Khan A Ghosh A Keshari R Bhatt M. Responsive nanostructure for targeted drug delivery. J Nanotheranostics (2023) 4(1):55–85. doi: 10.3390/jnt4010004

[49] Matea CT Mocan T Tabaran F Pop T Mosteanu O Puia C. Quantum dots in imaging, drug delivery and sensor applications. Int J Nanomed (2017) 12:5421–31. doi: 10.2147/ijn.S138624 PMC5546783 28814860

[50] Gidwani B Sahu V Shukla SS Pandey R Joshi V Jain VK. Quantum dots: Prospectives, toxicity, advances and applications. J Drug Delivery Sci Technol (2021) 61:102308. doi: 10.1016/j.jddst.2020.102308

[51] Zhao C Song X Liu Y Fu Y Ye L Wang N. Synthesis of graphene quantum dots and their applications in drug delivery. J Nanobiotechnol (2020) 18:142. doi: 10.1186/s12951-020-00698-z PMC7532648 33008457

[52] Siddique S Chow JCL. Gold nanoparticles for drug delivery and cancer therapy. Appl Sci (2020) 10(11):3824. doi: 10.3390/app10113824

[53] Kong FY Zhang JW Li RF Wang ZX Wang WJ Wang W. Unique roles of gold nanoparticles in drug delivery, targeting and imaging applications. Molecules (2017) 22(9):1445. doi: 10.3390/molecules22091445 28858253 PMC6151763

[54] Dadfar SM Roemhild K Drude NI von Stillfried S Knüchel R Kiessling F. Iron oxide nanoparticles: Diagnostic, therapeutic and theranostic applications. Advanced Drug delivery Rev (2019) 138:302–25. doi: 10.1016/j.addr.2019.01.005 PMC7115878 30639256

[55] Wahajuddin Arora S. Superparamagnetic iron oxide nanoparticles: magnetic nanoplatforms as drug carriers. Int J nanomedicine. (2012) 7:3445–71. doi: 10.2147/ijn.S30320 PMC3405876 22848170

[56] Chee CF Leo BF Lai CW. 37 - Superparamagnetic iron oxide nanoparticles for drug delivery. In: Inamuddin, Asiri AM Mohammad A, editors. Applications of nanocomposite materials in drug delivery. Duxford, UK: Woodhead Publishing (2018) p. 861–903.

[57] Han FY Thurecht KJ Whittaker AK Smith MT. Bioerodable PLGA-based microparticles for producing sustained-release drug formulations and strategies for improving drug loading. Front Pharmacol (2016) 7:185. doi: 10.3389/fphar.2016.00185 27445821 PMC4923250

[58] Astete CE Sabliov CM. Synthesis and characterization of PLGA nanoparticles. J biomaterials Sci Polymer edition. (2006) 17(3):247–89. doi: 10.1163/156856206775997322 16689015

[59] Sinha B Müller RH Möschwitzer JP. Bottom-up approaches for preparing drug nanocrystals: formulations and factors affecting particle size. Int J pharmaceutics. (2013) 453(1):126–41. doi: 10.1016/j.ijpharm.2013.01.019 23333709

[60] Kumar S Bhushan P Bhattacharya S. Fabrication of nanostructures with bottom-up approach and their utility in diagnostics, therapeutics, and others. Environmental Chem Med Sensors. (2017) 18:167–98. doi: 10.1007/978-981-10-7751-7_8

[61] Krishnaswamy K Orsat V. Chapter 2 - sustainable delivery systems through green nanotechnology. In: Grumezescu AM, editor. Nano- and microscale drug delivery systems. Elsevier (2017). p. 17–32.

[62] Reis CP Neufeld RJ Ribeiro AJ Veiga F. Nanoencapsulation I. Methods for preparation of drug-loaded polymeric nanoparticles. Nanomedicine: nanotechnology biology Med (2006) 2(1):8–21. doi: 10.1016/j.nano.2005.12.003 17292111

[63] Kraft JC Freeling JP Wang Z Ho RJY. Emerging research and clinical development trends of liposome and lipid nanoparticle drug delivery systems. J Pharm Sci (2014) 103(1):29–52. doi: 10.1002/jps.23773 24338748 PMC4074410

[64] Danaei M Dehghankhold M Ataei S Hasanzadeh Davarani F Javanmard R Dokhani A. Impact of particle size and polydispersity index on the clinical applications of lipidic nanocarrier systems. Pharmaceutics. (2018) 10(2):57. doi: 10.3390/pharmaceutics10020057 29783687 PMC6027495

[65] Yu W Liu R Zhou Y Gao H. Size-tunable strategies for a tumor targeted drug delivery system. ACS Cent Sci (2020) 6(2):100–16. doi: 10.1021/acscentsci.9b01139 PMC7047275 32123729

[66] Hrkach J Von Hoff D Mukkaram Ali M Andrianova E Auer J Campbell T. Preclinical development and clinical translation of a PSMA-targeted docetaxel nanoparticle with a differentiated pharmacological profile. Sci Trans Med (2012) 4(128):128ra39. doi: 10.1126/scitranslmed.3003651 22491949

[67] Mangadlao JD Wang X McCleese C Escamilla M Ramamurthy G Wang Z. Prostate-specific membrane antigen targeted gold nanoparticles for theranostics of prostate cancer. ACS Nano. (2018) 12(4):3714–25. doi: 10.1021/acsnano.8b00940 PMC6392200 29641905

[68] Lee JH Yeo Y. Controlled drug release from pharmaceutical nanocarriers. Chem Eng science. (2015) 125:75–84. doi: 10.1016/j.ces.2014.08.046 PMC4322773 25684779

[69] Kaushik N Borkar SB Nandanwar SK Panda PK Choi EH Kaushik NK. Nanocarrier cancer therapeutics with functional stimuli-responsive mechanisms. J nanobiotechnology. (2022) 20(1):152. doi: 10.1186/s12951-022-01364-2 35331246 PMC8944113

[70] Kinnear C Moore TL Rodriguez-Lorenzo L Rothen-Rutishauser B Petri-Fink A. Form follows function: nanoparticle shape and its implications for nanomedicine. Chem Rev (2017) 117(17):11476–521. doi: 10.1021/acs.chemrev.7b00194 28862437

[71] Lesniak WG Boinapally S Banerjee SR Behnam Azad B Foss CA Shen C. Evaluation of PSMA-targeted PAMAM dendrimer nanoparticles in a murine model of prostate cancer. Mol pharmaceutics. (2019) 16(6):2590–604. doi: 10.1021/acs.molpharmaceut.9b00181 31002252

[72] Flores O Santra S Kaittanis C Bassiouni R Khaled AS Khaled AR. PSMA-targeted theranostic nanocarrier for prostate cancer. Theranostics. (2017) 7(9):2477–94. doi: 10.7150/thno.18879 PMC5525751 28744329

[73] Afsharzadeh M Hashemi M Babaei M Abnous K Ramezani M. PEG-PLA nanoparticles decorated with small-molecule PSMA ligand for targeted delivery of galbanic acid and docetaxel to prostate cancer cells. J Cell Physiol (2020) 235(5):4618–30. doi: 10.1002/jcp.29339 31674023

[74] Meher N Ashley GW Bidkar AP Dhrona S Fong C Fontaine SD. Prostate-specific membrane antigen targeted deep tumor penetration of polymer nanocarriers. ACS Appl materials interfaces (2022) 14(45):50569–82. doi: 10.1021/acsami.2c15095 PMC9673064 36318757

[75] Cheng MHY Overchuk M Rajora MA Lou JWH Chen Y Pomper MG. Targeted theranostic (111)In/lu-nanotexaphyrin for SPECT imaging and photodynamic therapy. Mol Pharmaceutics (2021) 20(1):783. doi: 10.1021/acs.molpharmaceut.2c00905 36351175

[76] Essa D Kondiah PPD Kumar P Choonara YE. Design of chitosan-coated, quercetin-loaded PLGA nanoparticles for enhanced PSMA-specific activity on lnCap prostate cancer cells. Biomedicines (2023) 11(4):1201. doi: 10.3390/biomedicines11041201 37189819 PMC10136298

[77] Wong P Li L Chea J Delgado MK Crow D Poku E. PET imaging of (64)Cu-DOTA-scFv-anti-PSMA lipid nanoparticles (LNPs): Enhanced tumor targeting over anti-PSMA scFv or untargeted LNPs. Nucl Med Biol (2017) 47:62–8. doi: 10.1016/j.nucmedbio.2017.01.004 PMC5348925 28126683

[78] Tian JY Chi CL Bian G Xing D Guo FJ Wang XQ. PSMA conjugated combinatorial liposomal formulation encapsulating genistein and plumbagin to induce apoptosis in prostate cancer cells. Colloids surfaces B Biointerfaces. (2021) 203:111723. doi: 10.1016/j.colsurfb.2021.111723 33839474

[79] Rivero-Buceta E Vidaurre-Agut C Vera-Donoso CD Benlloch JM Moreno-Manzano V Botella P. PSMA-targeted mesoporous silica nanoparticles for selective intracellular delivery of docetaxel in prostate cancer cells. ACS Omega (2019) 4(1):1281–91. doi: 10.1021/acsomega.8b02909

[80] Lankoff A Czerwińska M Walczak R Karczmarczyk U Tomczyk K Brzóska K. Design and evaluation of (223)Ra-labeled and anti-PSMA targeted naA nanozeolites for prostate cancer therapy-part II. Toxicity, pharmacokinetics and biodistribution. Int J Mol Sci (2021) 22(11):5702. doi: 10.3390/ijms22115702 34071854 PMC8198605

[81] Liolios C Koutsikou TS Salvanou EA Kapiris F Machairas E Stampolaki M. Synthesis and *in vitro* proof-of-concept studies on bispecific iron oxide magnetic nanoparticles targeting PSMA and GRP receptors for PET/MR imaging of prostate cancer. Int J pharmaceutics. (2022) 624:122008. doi: 10.1016/j.ijpharm.2022.122008 35820513

[82] Comparetti EJ Romagnoli GG Gorgulho CM Pedrosa VA Kaneno R. Anti-PSMA monoclonal antibody increases the toxicity of paclitaxel carried by carbon nanotubes. Materials Sci Eng C Materials Biol applications. (2020) 116:111254. doi: 10.1016/j.msec.2020.111254 32806261

[83] Dostalova S Polanska H Svobodova M Balvan J Krystofova O Haddad Y. Prostate-specific membrane antigen-targeted site-directed antibody-conjugated apoferritin nanovehicle favorably influences *in vivo* side effects of doxorubicin. Sci Rep (2018) 8(1):8867. doi: 10.1038/s41598-018-26772-z 29891921 PMC5995913

[84] Behnam Azad B Banerjee SR Pullambhatla M Lacerda S Foss CA Wang Y. Evaluation of a PSMA-targeted BNF nanoparticle construct. Nanoscale. (2015) 7(10):4432–42. doi: 10.1039/c4nr06069e PMC5572130 25675333

[85] Meher N Seo K Wang S Bidkar AP Fogarty M Dhrona S. Synthesis and preliminary biological assessment of carborane-loaded theranostic nanoparticles to target prostate-specific membrane antigen. ACS Appl materials interfaces. (2021) 13(46):54739–52. doi: 10.1021/acsami.1c16383 PMC12490465 34752058

[86] Wu M Zhao H Guo L Wang Y Song J Zhao X. Ultrasound-mediated nanobubble destruction (UMND) facilitates the delivery of A10-3.2 aptamer targeted and siRNA-loaded cationic nanobubbles for therapy of prostate cancer. Drug delivery. (2018) 25(1):226–40. doi: 10.1080/10717544.2017.1422300 PMC6058493 29313393

[87] Saltman A Zegar J Haj-Hamed M Verma S Sidana A. Prostate cancer biomarkers and multiparametric MRI: is there a role for both in prostate cancer management? Ther Adv Urol (2021) 13:1756287221997186. doi: 10.1177/1756287221997186 33737957 PMC7934039

[88] Würnschimmel C Chandrasekar T Hahn L Esen T Shariat SF Tilki D. MRI as a screening tool for prostate cancer: current evidence and future challenges. World J Urol (2023) 41(4):921–8. doi: 10.1007/s00345-022-03947-y PMC10160206 35226140

[89] Rouvière O Puech P Renard-Penna R Claudon M Roy C Mège-Lechevallier F. Use of prostate systematic and targeted biopsy on the basis of multiparametric MRI in biopsy-naive patients (MRI-FIRST): a prospective, multicentre, paired diagnostic study. Lancet Oncol (2019) 20(1):100–9. doi: 10.1016/s1470-2045(18)30569-2 30470502

[90] Mastrogiacomo S Dou W Jansen JA Walboomers XF. Magnetic resonance imaging of hard tissues and hard tissue engineered bio-substitutes. Mol Imaging Biol (2019) 21(6):1003–19. doi: 10.1007/s11307-019-01345-2 30989438

[91] Beaton L Bandula S Gaze MN Sharma RA. How rapid advances in imaging are defining the future of precision radiation oncology. Br J cancer. (2019) 120(8):779–90. doi: 10.1038/s41416-019-0412-y PMC6474267 30911090

[92] Thariat J Hannoun-Levi JM Sun Myint A Vuong T Gérard JP. Past, present, and future of radiotherapy for the benefit of patients. Nat Rev Clin Oncol (2013) 10(1):52–60. doi: 10.1038/nrclinonc.2012.203 23183635

[93] Muralidhar A Potluri HK Jaiswal T McNeel DG. Targeted radiation and immune therapies-advances and opportunities for the treatment of prostate cancer. Pharmaceutics (2023) 15(1):252. doi: 10.3390/pharmaceutics15010252 36678880 PMC9863141

[94] Xiao YD Paudel R Liu J Ma C Zhang ZS Zhou SK. MRI contrast agents: Classification and application (Review). Int J Mol Med (2016) 38(5):1319–26. doi: 10.3892/ijmm.2016.2744 27666161

[95] Li X Sun Y Ma L Liu G Wang Z. The renal clearable magnetic resonance imaging contrast agents: state of the art and recent advances. Molecules (2020) 25(21):5072. doi: 10.3390/molecules25215072 33139643 PMC7662352

[96] Wahsner J Gale EM Rodríguez-Rodríguez A Caravan P. Chemistry of MRI contrast agents: current challenges and new frontiers. Chem Rev (2019) 119(2):957–1057. doi: 10.1021/acs.chemrev.8b00363 30350585 PMC6516866

[97] Zhu D Liu F Ma L Liu D Wang Z. Nanoparticle-based systems for T(1)-weighted magnetic resonance imaging contrast agents. Int J Mol Sci (2013) 14(5):10591–607. doi: 10.3390/ijms140510591 PMC3676856 23698781

[98] Miao Y Xie Q Zhang H Cai J Liu X Jiao J. Composition-Tunable Ultrasmall Manganese Ferrite Nanoparticles: Insights into their *In Vivo* T(1) Contrast Efficacy. Theranostics. (2019) 9(6):1764–76. doi: 10.7150/thno.31233 PMC6485191 31037137

[99] Cai X Zhu Q Zeng Y Zeng Q Chen X Zhan Y. Manganese oxide nanoparticles as MRI contrast agents in tumor multimodal imaging and therapy. Int J nanomedicine. (2019) 14:8321–44. doi: 10.2147/ijn.S218085 PMC6814316 31695370

[100] Li J Wu C Hou P Zhang M Xu K. One-pot preparation of hydrophilic manganese oxide nanoparticles as T(1) nano-contrast agent for molecular magnetic resonance imaging of renal carcinoma *in vitro* and *in vivo* . Biosensors bioelectronics (2018) 102:1–8. doi: 10.1016/j.bios.2017.10.047 29101783

[101] Lai J Wang T Wang H Shi F Gu W Ye L. MnO nanoparticles with unique excitation-dependent fluorescence for multicolor cellular imaging and MR imaging of brain glioma. Mikrochimica Acta (2018) 185(4):244. doi: 10.1007/s00604-018-2779-5 29610993

[102] Chen SH Huang LY Huang B Zhang M Li H Pang DW. Ultrasmall mnSe nanoparticles as T(1)-MRI contrast agents for *in vivo* tumor imaging. ACS Appl materials interfaces. (2022) 14(9):11167–76. doi: 10.1021/acsami.1c25101 35226454

[103] Li Y Zhao X Liu X Cheng K Han X Zhang Y. A bioinspired nanoprobe with multilevel responsive T(1) -weighted MR signal-amplification illuminates ultrasmall metastases. Advanced materials (Deerfield Beach Fla). (2020) 32(4):e1906799. doi: 10.1002/adma.201906799 31799765

[104] Wang J Li L Li Y Liu L Li J Li X. PSMA1-mediated ultrasmall gold nanoparticles facilitate tumor targeting and MR/CT/NIRF multimodal detection of early-stage prostate cancer. Nanomedicine: nanotechnology biology Med (2023) 47:102617. doi: 10.1016/j.nano.2022.102617 36280043

[105] Banerjee SR Ngen EJ Rotz MW Kakkad S Lisok A Pracitto R. Synthesis and evaluation of gd(III) -based magnetic resonance contrast agents for molecular imaging of prostate-specific membrane antigen. Angewandte Chemie (International ed English). (2015) 54(37):10778–82. doi: 10.1002/anie.201503417 PMC4729199 26212031

[106] Zhu Y Sun Y Chen Y Liu W Jiang J Guan W. *In vivo* molecular MRI imaging of prostate cancer by targeting PSMA with polypeptide-labeled superparamagnetic iron oxide nanoparticles. Int J Mol Sci (2015) 16(5):9573–87. doi: 10.3390/ijms16059573 PMC4463605 25927579

[107] Tse BW Cowin GJ Soekmadji C Jovanovic L Vasireddy RS Ling MT. PSMA-targeting iron oxide magnetic nanoparticles enhance MRI of preclinical prostate cancer. Nanomedicine (London England). (2015) 10(3):375–86. doi: 10.2217/nnm.14.122 25407827

[108] Ngen EJ Benham Azad B Boinapally S Lisok A Brummet M Jacob D. MRI assessment of prostate-specific membrane antigen (PSMA) targeting by a PSMA-targeted magnetic nanoparticle: potential for image-guided therapy. Mol pharmaceutics. (2019) 16(5):2060–8. doi: 10.1021/acs.molpharmaceut.9b00036 30912947

[109] Wang R Wang L Chen Y Xie Y He M Zhu Y. Biogenic gas vesicles for ultrasound imaging and targeted therapeutics. Curr medicinal Chem (2022) 29(8):1316–30. doi: 10.2174/0929867328666210705145642 34225604

[110] Mitterberger M Horninger W Aigner F Pinggera GM Steppan I Rehder P. Ultrasound of the prostate. Cancer imaging: Off Publ Int Cancer Imaging Society. (2010) 10(1):40–8. doi: 10.1102/1470-7330.2010.0004 PMC2842183 20199941

[111] Borden M Sirsi S. Ultrasound imaging: better contrast with vesicles. Nat nanotechnology. (2014) 9(4):248–9. doi: 10.1038/nnano.2014.68 24694873

[112] Shapiro MG Goodwill PW Neogy A Yin M Foster FS Schaffer DV. Biogenic gas nanostructures as ultrasonic molecular reporters. Nat nanotechnology. (2014) 9(4):311–6. doi: 10.1038/nnano.2014.32 PMC4023545 24633522

[113] Fan X Guo Y Wang L Xiong X Zhu L Fang K. Diagnosis of prostate cancer using anti-PSMA aptamer A10-3.2-oriented lipid nanobubbles. Int J nanomedicine (2016) 11:3939–50. doi: 10.2147/ijn.S112951 PMC4990382 27574424

[114] Wu M Wang Y Wang Y Zhang M Luo Y Tang J. Paclitaxel-loaded and A10-3.2 aptamer-targeted poly(lactide-co-glycolic acid) nanobubbles for ultrasound imaging and therapy of prostate cancer. Int J nanomedicine (2017) 12:5313–30. doi: 10.2147/ijn.S136032 PMC5536230 28794625

[115] Riksen JJM Nikolaev AV van Soest G. Photoacoustic imaging on its way toward clinical utility: a tutorial review focusing on practical application in medicine. J Biomed optics. (2023) 28(12):121205. doi: 10.1117/1.Jbo.28.12.121205 PMC10249868 37304059

[116] He C Zhu J Zhang H Qiao R Zhang R. Photoacoustic imaging probes for theranostic applications. Biosensors (2022) 12(11):947. doi: 10.3390/bios12110947 36354456 PMC9688356

[117] Steinberg I Huland DM Vermesh O Frostig HE Tummers WS Gambhir SS. Photoacoustic clinical imaging. Photoacoustics. (2019) 14:77–98. doi: 10.1016/j.pacs.2019.05.001 31293884 PMC6595011

[118] Qiu T Lan Y Gao W Zhou M Liu S Huang W. Photoacoustic imaging as a highly efficient and precise imaging strategy for the evaluation of brain diseases. Quantitative Imaging Med surgery. (2021) 11(5):2169–86. doi: 10.21037/qims-20-845 PMC8047382 33936997

[119] Alchera E Monieri M Maturi M Locatelli I Locatelli E Tortorella S. Early diagnosis of bladder cancer by photoacoustic imaging of tumor-targeted gold nanorods. Photoacoustics. (2022) 28:100400. doi: 10.1016/j.pacs.2022.100400 36386292 PMC9649962

[120] Wang Y Lan M Shen D Fang K Zhu L Liu Y. Targeted nanobubbles carrying indocyanine green for ultrasound, photoacoustic and fluorescence imaging of prostate cancer. Int J nanomedicine. (2020) 15:4289–309. doi: 10.2147/ijn.S243548 PMC7306459 32606678

[121] Kim D Han W Chang JH Lee HJ. PMP (Porphyrin-micelle-PSMA) nanoparticles for photoacoustic and ultrasound signal amplification in mouse prostate cancer xenografts. Pharmaceutics (2021) 13(10):1636. doi: 10.3390/pharmaceutics13101636 34683929 PMC8537944

[122] Machulkin AE Uspenskaya AA Zyk NY Nimenko EA Ber AP Petrov SA. PSMA-targeted small-molecule docetaxel conjugate: Synthesis and preclinical evaluation. Eur J medicinal Chem (2022) 227:113936. doi: 10.1016/j.ejmech.2021.113936 34717125

[123] de Wit R de Bono J Sternberg CN Fizazi K Tombal B Wülfing C. Cabazitaxel versus abiraterone or enzalutamide in metastatic prostate cancer. New Engl J Med (2019) 381(26):2506–18. doi: 10.1056/NEJMoa1911206 31566937

[124] Stenzl A Dunshee C De Giorgi U Alekseev B Iguchi T Szmulewitz RZ. Effect of enzalutamide plus androgen deprivation therapy on health-related quality of life in patients with metastatic hormone-sensitive prostate cancer: an analysis of the ARCHES randomised, placebo-controlled, phase 3 study. Eur urology. (2020) 78(4):603–14. doi: 10.1016/j.eururo.2020.03.019 32336645

[125] Hikita K Honda M Shimizu R Teraoka S Kawamoto B Yumioka T. Efficacy of combination chemotherapy with docetaxel, estramustine and carboplatin in men with castration-resistant prostate cancer. Cancer diagnosis prognosis. (2021) 1(5):451–7. doi: 10.21873/cdp.10060 PMC8962872 35403156

[126] Sweeney CJ Chen Y-H Carducci M Liu G Jarrard DF Eisenberger M. Chemohormonal therapy in metastatic. Hormone-Sensitive Prostate Cancer. (2015) 373(8):737–46. doi: 10.1056/NEJMoa1503747 PMC4562797 26244877

[127] Xu W Ding J Xiao C Li L Zhuang X Chen X. Versatile preparation of intracellular-acidity-sensitive oxime-linked polysaccharide-doxorubicin conjugate for Malignancy therapeutic. Biomaterials. (2015) 54:72–86. doi: 10.1016/j.biomaterials.2015.03.021 25907041

[128] Ding J Xiao C Li Y Cheng Y Wang N He C. Efficacious hepatoma-targeted nanomedicine self-assembled from galactopeptide and doxorubicin driven by two-stage physical interactions. J Controlled Release (2013) 169(3):193–203. doi: 10.1016/j.jconrel.2012.12.006 23247039

[129] Hu T Gong H Xu J Huang Y Wu F He Z. Nanomedicines for overcoming cancer drug resistance. Pharmaceutics (2022) 14(8):1606. doi: 10.3390/pharmaceutics14081606 36015232 PMC9412887

[130] Wei G Wang Y Yang G Wang Y Ju R. Recent progress in nanomedicine for enhanced cancer chemotherapy. Theranostics. (2021) 11(13):6370–92. doi: 10.7150/thno.57828 PMC8120226 33995663

[131] Zhang X Wang Y Wei G Zhao J Yang G Zhou S. Stepwise dual targeting and dual responsive polymer micelles for mitochondrion therapy. J Controlled release: Off J Controlled Release Society. (2020) 322:157–69. doi: 10.1016/j.jconrel.2020.03.011 32169533

[132] Dai J Wu Y Chen Z Xiao L Zhang W Cao Y. Sonosensitive phase-changeable nanoparticle mediated enhanced chemotherapy in prostate cancer by low-intensity focused ultrasound. Int J Mol Sci (2023) 24(1):825. doi: 10.3390/ijms24010825 36614265 PMC9821565

[133] Tannock IF de Wit R Berry WR Horti J Pluzanska A Chi KN. Docetaxel plus prednisone or mitoxantrone plus prednisone for advanced prostate cancer. New Engl J Med (2004) 351(15):1502–12. doi: 10.1056/NEJMoa040720 15470213

[134] Petrylak DP Tangen CM Hussain MH Lara PN Jr. Jones JA Taplin ME. Docetaxel and estramustine compared with mitoxantrone and prednisone for advanced refractory prostate cancer. New Engl J Med (2004) 351(15):1513–20. doi: 10.1056/NEJMoa041318 15470214

[135] Saniee F Shabani Ravari N Goodarzi N Amini M Atyabi F Saeedian Moghadam E. Glutamate-urea-based PSMA-targeted PLGA nanoparticles for prostate cancer delivery of docetaxel. Pharm Dev technology. (2021) 26(4):381–9. doi: 10.1080/10837450.2021.1875238 33538232

[136] Nagesh PKB Johnson NR Boya VKN Chowdhury P Othman SF Khalilzad-Sharghi V. PSMA targeted docetaxel-loaded superparamagnetic iron oxide nanoparticles for prostate cancer. Colloids surfaces B Biointerfaces. (2016) 144:8–20. doi: 10.1016/j.colsurfb.2016.03.071 27058278 PMC5100699

[137] Von Hoff DD Mita MM Ramanathan RK Weiss GJ Mita AC LoRusso PM. Phase I study of PSMA-targeted docetaxel-containing nanoparticle BIND-014 in patients with advanced solid tumors. Clin Cancer research: an Off J Am Assoc Cancer Res (2016) 22(13):3157–63. doi: 10.1158/1078-0432.Ccr-15-2548 26847057

[138] Oudard S Fizazi K Sengeløv L Daugaard G Saad F Hansen S. Cabazitaxel versus docetaxel as first-line therapy for patients with metastatic castration-resistant prostate cancer: A randomized phase III trial-FIRSTANA. J Clin oncology: Off J Am Soc Clin Oncol (2017) 35(28):3189–97. doi: 10.1200/jco.2016.72.1068 28753384

[139] Cohen L Assaraf YG Livney YD. Novel selectively targeted multifunctional nanostructured lipid carriers for prostate cancer treatment. Pharmaceutics (2021) 14(1):88. doi: 10.3390/pharmaceutics14010088 35056984 PMC8781189

[140] Wang C Zhu X Hong JC Zheng D. Artificial intelligence in radiotherapy treatment planning: present and future. Technol Cancer Res Treat (2019) 18:1533033819873922. doi: 10.1177/1533033819873922 31495281 PMC6732844

[141] Thoms J Goda JS Zlotta AR Fleshner NE van der Kwast TH Supiot S. Neoadjuvant radiotherapy for locally advanced and high-risk prostate cancer. Nat Rev Clin Oncol (2011) 8(2):107–13. doi: 10.1038/nrclinonc.2010.207 21178999

[142] Podder TK Fredman ET Ellis RJ. Advances in radiotherapy for prostate cancer treatment. Adv Exp Med Biol (2018) 1096:31–47. doi: 10.1007/978-3-319-99286-0_2 30324346

[143] Gong L Zhang Y Liu C Zhang M Han S. Application of radiosensitizers in cancer radiotherapy. Int J nanomedicine. (2021) 16:1083–102. doi: 10.2147/ijn.S290438 PMC7886779 33603370

[144] Wang H Mu X He H Zhang X-D. Cancer radiosensitizers. Trends Pharmacol Sci (2018) 39(1):24–48. doi: 10.1016/j.tips.2017.11.003 29224916

[145] Ma N Liu P He N Gu N Wu FG Chen Z. Action of gold nanospikes-based nanoradiosensitizers: cellular internalization, radiotherapy, and autophagy. ACS Appl materials interfaces. (2017) 9(37):31526–42. doi: 10.1021/acsami.7b09599 28816044

[146] Singh P Pandit S Mokkapati VRSS Garg A Ravikumar V Mijakovic I. Gold nanoparticles in diagnostics and therapeutics for human cancer. Int J Mol Sci (2018) 19(7):1979. doi: 10.3390/ijms19071979 29986450 PMC6073740

[147] Rosa S Connolly C Schettino G Butterworth KT Prise KM. Biological mechanisms of gold nanoparticle radiosensitization. Cancer nanotechnology. (2017) 8(1):2. doi: 10.1186/s12645-017-0026-0 28217176 PMC5288470

[148] Her S Jaffray DA Allen C. Gold nanoparticles for applications in cancer radiotherapy: Mechanisms and recent advancements. Advanced Drug delivery Rev (2017) 109:84–101. doi: 10.1016/j.addr.2015.12.012 26712711

[149] Luo D Wang X Zeng S Ramamurthy G Burda C Basilion JP. Targeted gold nanocluster-enhanced radiotherapy of prostate cancer. Small (Weinheim an der Bergstrasse Germany). (2019) 15(34):e1900968. doi: 10.1002/smll.201900968 31265213 PMC6707872

[150] Luo D Johnson A Wang X Li H Erokwu BO Springer S. Targeted radiosensitizers for MR-guided radiation therapy of prostate cancer. Nano letters. (2020) 20(10):7159–67. doi: 10.1021/acs.nanolett.0c02487 PMC9109254 32845644

[151] Merkel C Whicher CH Bomanji J Herrmann K Ćwikła J Jervis N. Realising the potential of radioligand therapy: policy solutions for the barriers to implementation across Europe. Eur J Nucl Med Mol imaging. (2020) 47(6):1335–9. doi: 10.1007/s00259-020-04745-7 PMC7188707 32170345

[152] Zhao J Zhou M Li C. Synthetic nanoparticles for delivery of radioisotopes and radiosensitizers in cancer therapy. Cancer nanotechnology. (2016) 7(1):9. doi: 10.1186/s12645-016-0022-9 27909463 PMC5112292

[153] Ju Z Wang Z Wang L Li J Wu Z Li X. Experimental study on radiation damage of(125)I seeds implanted in canine gastric wall tissue. J Cancer Res Ther (2020) 16(2):203–8. doi: 10.4103/jcrt.JCRT_544_19 32474502

[154] Yavuz S Puckett Y. Iodine-131 uptake study. StatPearls. Treasure Island (FL: StatPearls PublishingCopyright © 2023, StatPearls Publishing LLC (2023). 32644709

[155] Lepareur N Lacœuille F Bouvry C Hindré F Garcion E Chérel M. Rhenium-188 labeled radiopharmaceuticals: current clinical applications in oncology and promising perspectives. Front Med (2019) 6:132. doi: 10.3389/fmed.2019.00132 PMC6587137 31259173

[156] Yong KJ Milenic DE Baidoo KE Brechbiel MW. Mechanisms of cell killing response from low linear energy transfer (LET) radiation originating from (177)Lu radioimmunotherapy targeting disseminated intraperitoneal tumor xenografts. Int J Mol Sci (2016) 17(5):736. doi: 10.3390/ijms17050736 27196891 PMC4881558

[157] Giesel FL Adeberg S Syed M Lindner T Jiménez-Franco LD Mavriopoulou E. FAPI-74 PET/CT using either (18)F-alF or cold-kit (68)Ga labeling: biodistribution, radiation dosimetry, and tumor delineation in lung cancer patients. J Nucl medicine: Off publication Soc Nucl Med (2021) 62(2):201–7. doi: 10.2967/jnumed.120.245084 PMC8679591 32591493

[158] Salih S Alkatheeri A Alomaim W Elliyanti A. Radiopharmaceutical treatments for cancer therapy, radionuclides characteristics, applications, and challenges. Molecules (2022) 27(16):5231. doi: 10.3390/molecules27165231 36014472 PMC9415873

[159] Benešová M Schäfer M Bauder-Wüst U Afshar-Oromieh A Kratochwil C Mier W. Preclinical evaluation of a tailor-made DOTA-conjugated PSMA inhibitor with optimized linker moiety for imaging and endoradiotherapy of prostate cancer. J Nucl Med (2015) 56(6):914–20. doi: 10.2967/jnumed.114.147413%J 25883127

[160] Weineisen M Schottelius M Simecek J Baum RP Yildiz A Beykan S. 68Ga- and 177Lu-labeled PSMA I&T: optimization of a PSMA-targeted theranostic concept and first proof-of-concept human studies. J Nucl Med (2015) 56(8):1169–76. doi: 10.2967/jnumed.115.158550 26089548

[161] Sartor O de Bono J Chi KN Fizazi K Herrmann K Rahbar K. Lutetium-177–PSMA-617 for metastatic castration-resistant prostate cancer. N Engl J Med (2021) 385(12):1091–103. doi: 10.1056/NEJMoa2107322 PMC8446332 34161051

[162] Kratochwil C Giesel FL Stefanova M Benešová M Bronzel M Afshar-Oromieh A. PSMA-targeted radionuclide therapy of metastatic castration-resistant prostate cancer with 177Lu-labeled PSMA-617. J Nucl medicine: Off publication Soc Nucl Med (2016) 57(8):1170–6. doi: 10.2967/jnumed.115.171397 26985056

[163] Ahmadzadehfar H Eppard E Kürpig S Fimmers R Yordanova A Schlenkhoff CD. Therapeutic response and side effects of repeated radioligand therapy with 177Lu-PSMA-DKFZ-617 of castrate-resistant metastatic prostate cancer. Oncotarget. (2016) 7(11):12477–88. doi: 10.18632/oncotarget.7245 PMC4914299 26871285

[164] Rahbar K Schmidt M Heinzel A Eppard E Bode A Yordanova A. Response and tolerability of a single dose of 177Lu-PSMA-617 in patients with metastatic castration-resistant prostate cancer: A multicenter retrospective analysis. J Nucl medicine: Off publication Soc Nucl Med (2016) 57(9):1334–8. doi: 10.2967/jnumed.116.173757 27056618

[165] Rahbar K Bode A Weckesser M Avramovic N Claesener M Stegger L. Radioligand therapy with 177Lu-PSMA-617 as A novel therapeutic option in patients with metastatic castration resistant prostate cancer. Clin Nucl Med (2016) 41(7):522–8. doi: 10.1097/rlu.0000000000001240 27088387

[166] Baum RP Kulkarni HR Schuchardt C Singh A Wirtz M Wiessalla S. 177Lu-labeled prostate-specific membrane antigen radioligand therapy of metastatic castration-resistant prostate cancer: safety and efficacy. J Nucl medicine: Off publication Soc Nucl Med (2016) 57(7):1006–13. doi: 10.2967/jnumed.115.168443 26795286

[167] Rahbar K Ahmadzadehfar H Kratochwil C Haberkorn U Schäfers M Essler M. German multicenter study investigating 177Lu-PSMA-617 radioligand therapy in advanced prostate cancer patients. J Nucl Med(2017) 58(1):85–90. doi: 10.2967/jnumed.116.183194 27765862

[168] Hofman MS Violet J Hicks RJ Ferdinandus J Thang SP Akhurst T. [(177)Lu]-PSMA-617 radionuclide treatment in patients with metastatic castration-resistant prostate cancer (LuPSMA trial): a single-centre, single-arm, phase 2 study. Lancet Oncol (2018) 19(6):825–33. doi: 10.1016/s1470-2045(18)30198-0 29752180

[169] Calais J Gafita A Eiber M Armstrong WR Gartmann J Thin P. Prospective phase 2 trial of PSMA-targeted molecular RadiothErapy with (177)Lu-PSMA-617 for metastatic castration-reSISTant Prostate Cancer (RESIST-PC): efficacy results of the UCLA cohort. J Nucl medicine: Off publication Soc Nucl Med (2021) 62(10):1440–6. doi: 10.2967/jnumed.121.261982 PMC8724893 34016732

[170] Boateng F Ngwa W. Delivery of nanoparticle-based radiosensitizers for radiotherapy applications. Int J Mol Sci (2019) 21(1):273. doi: 10.3390/ijms21010273 31906108 PMC6981554

[171] Song X Sun Z Li L Zhou L Yuan S. Application of nanomedicine in radiotherapy sensitization. Front Oncol (2023) 13:1088878. doi: 10.3389/fonc.2023.1088878 36874097 PMC9977159

[172] Czerwińska M Fracasso G Pruszyński M Bilewicz A Kruszewski M Majkowska-Pilip A. Design and evaluation of (223)Ra-labeled and anti-PSMA targeted naA nanozeolites for prostate cancer therapy-part I. Materials (Basel) (2020) 13(17):3875. doi: 10.3390/ma13173875 32887308 PMC7504699

[173] Agostinis P Berg K Cengel KA Foster TH Girotti AW Gollnick SO. Photodynamic therapy of cancer: an update. CA: Cancer J Clin (2011) 61(4):250–81. doi: 10.3322/caac.20114 PMC3209659 21617154

[174] Su Z Yang Z Xu Y Chen Y Yu Q. Apoptosis, autophagy, necroptosis, and cancer metastasis. Mol cancer. (2015) 14:48. doi: 10.1186/s12943-015-0321-5 25743109 PMC4343053

[175] Hu J Luo H Qu Q Liao X Huang C Chen J. Cell membrane-inspired polymeric vesicles for combined photothermal and photodynamic prostate cancer therapy. ACS Appl materials interfaces. (2020) 12(38):42511–20. doi: 10.1021/acsami.0c11636 32897691

[176] Kessel D. Photodynamic therapy: apoptosis, paraptosis and beyond. Apoptosis: an Int J programmed Cell death. (2020) 25(9-10):611–5. doi: 10.1007/s10495-020-01634-0 32888113

[177] Wilson BC Weersink RA. The yin and yang of PDT and PTT. Photochem photobiology. (2020) 96(2):219–31. doi: 10.1111/php.13184 31769516

[178] Davidson SR Weersink RA Haider MA Gertner MR Bogaards A Giewercer D. Treatment planning and dose analysis for interstitial photodynamic therapy of prostate cancer. Phys Med Biol (2009) 54(8):2293–313. doi: 10.1088/0031-9155/54/8/003 19305043

[179] Kannadorai RK Liu Q. Optimization in interstitial plasmonic photothermal therapy for treatment planning. Med physics. (2013) 40(10):103301. doi: 10.1118/1.4810935 24089931

[180] Li Z Nguyen L Bass DA Baran TM. Effects of patient-specific treatment planning on eligibility for photodynamic therapy of deep tissue abscess cavities: retrospective Monte Carlo simulation study. J Biomed Opt (2022) 27(8):083007. doi: 10.1117/1.Jbo.27.8.083007 35146973 PMC8831513

[181] Kim MM Penjweini R Zhu TC. Evaluation of singlet oxygen explicit dosimetry for predicting treatment outcomes of benzoporphyrin derivative monoacid ring A-mediated photodynamic therapy. J Biomed optics. (2017) 22(2):28002. doi: 10.1117/1.Jbo.22.2.028002 PMC5301138 28301655

[182] Egorov SY Kamalov VF Koroteev NI Krasnovsky AA Toleutaev BN Zinukov SV. Rise and decay kinetics of photosensitized singlet oxygen luminescence in water. Measurements with nanosecond time-correlated single photon counting technique. Chem Phys Lett (1989) 163(4):421–4. doi: 10.1016/0009-2614(89)85161-9

[183] Ferroni C Del Rio A Martini C Manoni E Varchi G. Light-induced therapies for prostate cancer treatment. Front Chem (2019) 7:719. doi: 10.3389/fchem.2019.00719 31737599 PMC6828976

[184] Harmatys KM Overchuk M Chen J Ding L Chen Y Pomper MG. Tuning pharmacokinetics to improve tumor accumulation of a prostate-specific membrane antigen-targeted phototheranostic agent. Bioconjugate Chem (2018) 29(11):3746–56. doi: 10.1021/acs.bioconjchem.8b00636 PMC6813806 30350576

[185] Overchuk M Damen MPF Harmatys KM Pomper MG Chen J Zheng G. Long-circulating prostate-specific membrane antigen-targeted NIR phototheranostic agent. Photochem photobiology. (2020) 96(3):718–24. doi: 10.1111/php.13181 31742696

[186] Wang X Tsui B Ramamurthy G Zhang P Meyers J Kenney ME. Theranostic agents for photodynamic therapy of prostate cancer by targeting prostate-specific membrane antigen. Mol Cancer Ther (2016) 15(8):1834–44. doi: 10.1158/1535-7163.Mct-15-0722 27297866

[187] Luo D Wang X Walker E Wang J Springer S Lou J. Nanoparticles yield increased drug uptake and therapeutic efficacy upon sequential near-infrared irradiation. ACS Nano. (2020) 14(11):15193–203. doi: 10.1021/acsnano.0c05425 PMC9109620 33090762

[188] Phan LMT Gul AR Le TN Kim MW Kailasa SK Oh KT. One-pot synthesis of carbon dots with intrinsic folic acid for synergistic imaging-guided photothermal therapy of prostate cancer cells. Biomaterials science. (2019) 7(12):5187–96. doi: 10.1039/c9bm01228a 31588457

[189] Dai L Shen G Wang Y Yang P Wang H Liu Z. PSMA-targeted melanin-like nanoparticles as a multifunctional nanoplatform for prostate cancer theranostics. J materials Chem B (2021) 9(4):1151–61. doi: 10.1039/d0tb02576c 33434248

[190] Xia L Meng X Wen L Zhou N Liu T Xu X. A highly specific multiple enhancement theranostic nanoprobe for PET/MRI/PAI image-guided radioisotope combined photothermal therapy in prostate cancer. Small (Weinheim an der Bergstrasse Germany). (2021) 17(21):e2100378. doi: 10.1002/smll.202100378 33870644

[191] Xiao L Wu Y Dai J Zhang W Cao Y. Laser-activated nanoparticles for ultrasound/photoacoustic imaging-guided prostate cancer treatment. Front bioengineering Biotechnol (2023) 11:1141984. doi: 10.3389/fbioe.2023.1141984 PMC10070956 37025361

[192] Sharma P Pachynski RK Narayan V Fléchon A Gravis G Galsky MD. Nivolumab plus ipilimumab for metastatic castration-resistant prostate cancer: preliminary analysis of patients in the checkMate 650 trial. Cancer Cell (2020) 38(4):489–99. doi: 10.1016/j.ccell.2020.08.007 32916128

[193] Perera MPJ Thomas PB Risbridger GP Taylor R Azad A Hofman MS. Chimeric antigen receptor T-cell therapy in metastatic castrate-resistant prostate cancer. Cancers (2022) 14(3):503. doi: 10.3390/cancers14030503 35158771 PMC8833489

[194] Anassi E Ndefo UA. Sipuleucel-T (provenge) injection: the first immunotherapy agent (vaccine) for hormone-refractory prostate cancer. P T: peer-reviewed J formulary management. (2011) 36(4):197–202. PMC3086121 21572775

[195] He M Zhang D Cao Y Chi C Zeng Z Yang X. Chimeric antigen receptor-modified T cells therapy in prostate cancer: A comprehensive review on the current state and prospects. Heliyon (2023) 9(8):e19147. doi: 10.1016/j.heliyon.2023.e19147 37664750 PMC10469587

[196] Cha HR Lee JH Ponnazhagan S. Revisiting immunotherapy: A focus on prostate cancer. Cancer Res (2020) 80(8):1615–23. doi: 10.1158/0008-5472.Can-19-2948 PMC7641094 32066566

[197] Liu GB Zhao L Zhang L Zhao KN. Virus, oncolytic virus and human prostate cancer. Curr Cancer Drug targets. (2017) 17(6):522–33. doi: 10.2174/1568009616666161216095308 27993115

[198] Zhang J Cunningham JJ Brown JS Gatenby RA. Integrating evolutionary dynamics into treatment of metastatic castrate-resistant prostate cancer. Nat Commun (2017) 8(1):1816. doi: 10.1038/s41467-017-01968-5 29180633 PMC5703947

[199] Moreira DM Howard LE Sourbeer KN Amarasekara HS Chow LC Cockrell DC. Predicting time from metastasis to overall survival in castration-resistant prostate cancer: results from SEARCH. Clin Genitourin Cancer. (2017) 15(1):60–66.e2. doi: 10.1016/j.clgc.2016.08.018 27692812 PMC5536956

[200] Pagotto S Simeone P Brocco D Catitti G De Bellis D Vespa S. CAR-T-derived extracellular vesicles: A promising development of CAR-T anti-tumor therapy. Cancers (2023) 15(4):1052. doi: 10.3390/cancers15041052 36831396 PMC9954490

[201] Junghans RP Ma Q Rathore R Gomes EM Bais AJ Lo AS. Phase I trial of anti-PSMA designer CAR-T cells in prostate cancer: possible role for interacting interleukin 2-T cell pharmacodynamics as a determinant of clinical response. Prostate. (2016) 76(14):1257–70. doi: 10.1002/pros.23214 27324746

[202] Hassani M Hajari Taheri F Sharifzadeh Z Arashkia A Hadjati J van Weerden WM. Engineered jurkat cells for targeting prostate-specific membrane antigen on prostate cancer cells by nanobody-based chimeric antigen receptor. Iranian Biomed J (2020) 24(2):81–8. doi: 10.29252/ibj.24.2.81 PMC6984713 31677604

[203] Liu JP Wang TT Wang DG Dong AJ Li YP Yu HJ. Smart nanoparticles improve therapy for drug-resistant tumors by overcoming pathophysiological barriers. Acta pharmacologica Sinica. (2017) 38(1):1–8. doi: 10.1038/aps.2016.84 27569390 PMC5220546

[204] Zhao J Zhang C Wang W Li C Mu X Hu K. Current progress of nanomedicine for prostate cancer diagnosis and treatment. Biomedicine pharmacotherapy = Biomedecine pharmacotherapie. (2022) 155:113714. doi: 10.1016/j.biopha.2022.113714 36150309

[205] Wang Q Alshaker H Böhler T Srivats S Chao Y Cooper C. Core shell lipid-polymer hybrid nanoparticles with combined docetaxel and molecular targeted therapy for the treatment of metastatic prostate cancer. Sci Rep (2017) 7(1):5901. doi: 10.1038/s41598-017-06142-x 28724986 PMC5517417

[206] Bockamp E Rosigkeit S Siegl D Schuppan D. Nano-enhanced cancer immunotherapy: immunology encounters nanotechnology. Cells. (2020) 9(9):2102. doi: 10.3390/cells9092102 32942725 PMC7565449

[207] Briukhovetska D Dörr J Endres S Libby P Dinarello CA Kobold S. Interleukins in cancer: from biology to therapy. Nat Rev Cancer. (2021) 21(8):481–99. doi: 10.1038/s41568-021-00363-z PMC8173513 34083781

[208] Berman E Noyman I Medvedovsky M Ekstein D Eyal S. Not your usual drug-drug interactions: Monoclonal antibody-based therapeutics may interact with antiseizure medications. Epilepsia. (2022) 63(2):271–89. doi: 10.1111/epi.17147 34967010

[209] Jin S Sun Y Liang X Gu X Ning J Xu Y. Emerging new therapeutic antibody derivatives for cancer treatment. Signal Transduction Targeted Ther (2022) 7(1):39. doi: 10.1038/s41392-021-00868-x PMC8821599 35132063

[210] Wang D Shao Y Zhang X Lu G Liu B. IL-23 and PSMA-targeted duo-CAR T cells in Prostate Cancer Eradication in a preclinical model. J Trans Med (2020) 18(1):23. doi: 10.1186/s12967-019-02206-w PMC6961333 31937346

[211] Sugimoto Y Hirota M Yoshikawa K Sumitomo M Nakamura K Ueda R. The therapeutic potential of a novel PSMA antibody and its IL-2 conjugate in prostate cancer. Anticancer Res (2014) 34(1):89–97. 24403448

[212] Yang Y Hong Y Cho E Kim GB Kim IS. Extracellular vesicles as a platform for membrane-associated therapeutic protein delivery. J extracellular vesicles. (2018) 7(1):1440131. doi: 10.1080/20013078.2018.1440131 29535849 PMC5844050

[213] Doyle LM Wang MZ. Overview of extracellular vesicles, their origin, composition, purpose, and methods for exosome isolation and analysis. Cells (2019) 8(7):727. doi: 10.3390/cells8070727 31311206 PMC6678302

[214] Hagaman DE Damasco JA Perez JVD Rojo RD Melancon MP. Recent advances in nanomedicine for the diagnosis and treatment of prostate cancer bone metastasis. Molecules (2021) 26(2):384. doi: 10.3390/molecules26020384 33450939 PMC7828457

[215] Wang Z Zhi K Ding Z Sun Y Li S Li M. Emergence in protein derived nanomedicine as anticancer therapeutics: More than a tour de force. Semin Cancer Biol (2021) 69:77–90. doi: 10.1016/j.semcancer.2019.11.012 31962173

[216] He B Sui X Yu B Wang S Shen Y Cong H. Recent advances in drug delivery systems for enhancing drug penetration into tumors. Drug delivery. (2020) 27(1):1474–90. doi: 10.1080/10717544.2020.1831106 PMC7594734 33100061

[217] Sun W Deng Y Zhao M Jiang Y Gou J Wang Y. Targeting therapy for prostate cancer by pharmaceutical and clinical pharmaceutical strategies. J Controlled release: Off J Controlled Release Society (2021) 333:41–64. doi: 10.1016/j.jconrel.2021.01.010 33450321

[218] Shirokii N Din Y Petrov I Seregin Y Sirotenko S Razlivina J. Quantitative prediction of inorganic nanomaterial cellular toxicity via machine learning. Small (2023) 19(19):e2207106. doi: 10.1002/smll.202207106 36772908

[219] Zaman MU Fatima N Zaman A Sajid M Zaman U Zaman S. Diagnostic challenges in prostate cancer and 68Ga-PSMA PET imaging: A game changer? Asian Pacific J Cancer prevention: APJCP (2017) 18(10):2625–8. doi: 10.22034/apjcp.2017.18.10.2625 PMC5747380 29072055

[220] Stefan AK Katharina S Clemens K Erik W Matthias FH Sonja K. Clinical outcome of PSMA-guided radiotherapy for patients with oligorecurrent prostate cancer. Eur J Nucl Med Mol imaging. (2021) 48(1):143–51. doi: 10.1007/s00259-020-04777-z PMC7835298 32405735

[221] Schepisi G Cursano MC Casadei C Menna C Altavilla A Lolli C. CAR-T cell therapy: a potential new strategy against prostate cancer. J immunotherapy cancer. (2019) 7(1):258. doi: 10.1186/s40425-019-0741-7 PMC6794851 31619289

[222] Benzon B Glavaris SA Simons BW Hughes RM Ghabili K Mullane P. Combining immune check-point blockade and cryoablation in an immunocompetent hormone sensitive murine model of prostate cancer. Prostate Cancer prostatic diseases. (2018) 21(1):126–36. doi: 10.1038/s41391-018-0035-z PMC5990963 29556048

